# MSC transplantation ameliorates depression in lupus by suppressing Th1 cell–shaped synaptic stripping

**DOI:** 10.1172/jci.insight.181885

**Published:** 2025-03-06

**Authors:** Xiaojuan Han, Dandan Wang, Liang Chen, Hua Song, Xiulan Zheng, Xin Zhang, Shengnan Zhao, Jun Liang, Tianshu Xu, Zhibin Hu, Lingyun Sun

**Affiliations:** 1 Department of Rheumatology and Immunology, Nanjing Drum Tower Hospital Clinical College of Nanjing Medical University, State Key Laboratory of Reproductive Medicine and Offspring Health, Nanjing Medical University, Nanjing, China.; 2 Department of Rheumatology and Immunology, Nanjing Drum Tower Hospital, the Affiliated Hospital of Nanjing University Medical School, Nanjing, China.; 3 Department of Gynecology, The First Affiliated Hospital of Nanjing Medical University, Nanjing, China.; 4 School of Pharmacy, Macau University of Science and Technology, Macau, China.; 5 Department of Traditional Chinese Medicine, Nanjing Drum Tower Hospital Clinical College of Nanjing University of Chinese Medicine, Nanjing, China.; 6 State Key Laboratory of Reproductive Medicine and Offspring Health, Nanjing Medical University, Nanjing, China.; 7 Department of Epidemiology, Center for Global Health, School of Public Health, Nanjing Medical University, Nanjing, China.; 8 Department of Rheumatology and Immunology, The First Affiliated Hospital of Anhui Medical University, Hefei, China.

**Keywords:** Autoimmunity, Inflammation, Neuroscience, Autoimmune diseases, Depression, Immunotherapy

## Abstract

Systemic lupus erythematosus (SLE), an autoimmune disease, can cause psychiatric disorders, particularly depression, via immune activation. Human umbilical cord mesenchymal stromal cell (hUCMSC) transplantation (MSCT) has been shown to ameliorate immune dysfunction in SLE by inducing immune tolerance. However, whether MSCT can relieve the depressive symptoms in SLE remains incompletely understood. Here, we demonstrate that MSCT relieved early-onset depression-like behavior in both genetically lupus-prone (MRL/lpr) and pristane-induced lupus mice by rescuing impaired hippocampal synaptic connectivity. Transplanted hUCMSCs targeted Th1 cell–derived IFN-γ to inhibit neuronal JAK/STAT1 signaling and downstream CCL8 expression, reducing phagocytic microglia apposition to alleviate synaptic engulfment and neurological dysfunction in young (8-week-old) lupus mice. Systemic delivery of exogenous IFN-γ blunted MSCT-mediated alleviation of synaptic loss and depressive behavior in lupus mice, suggesting that the IFN-γ/CCL8 axis may be an effective therapeutic target and that MSCT is a potential therapy for lupus-related depression. In summary, transplanted hUCMSCs can target systemic immunity to ameliorate psychiatric disorders by rescuing synaptic loss, highlighting the active role of neurons as intermediaries between systemic immunity and microglia in this process.

## Abstract 2

MSCT therapeutically suppresses T cell-shaped neuroinflammation and decreases neuron-coordinated synaptic stripping to ameliorate depression in lupus

## Graphical abstract

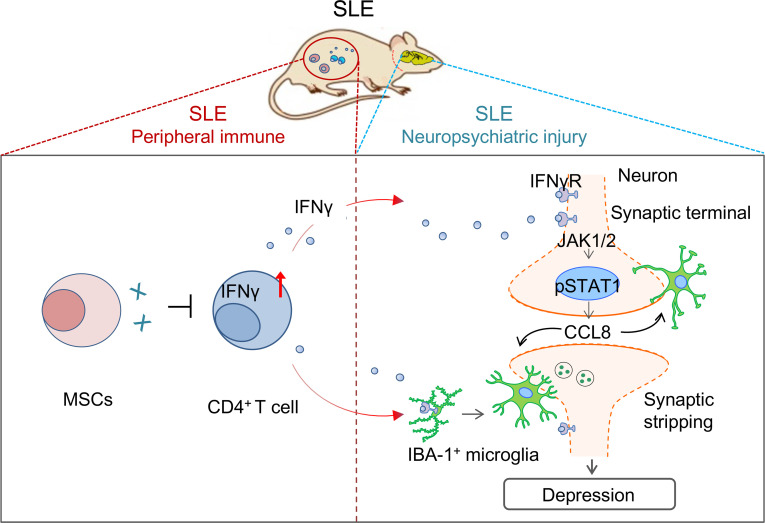

## Introduction

Systemic lupus erythematosus (SLE) is frequently associated with a range of diffuse central nervous system (CNS) disorders, termed CNS lupus or neuropsychiatric SLE (NPSLE), which can manifest as depression, anxiety, cognitive impairment, etc. (1). Depression is one of the most common mood disorders in patients with SLE and occurs early during the disease process (2, 3). Increased depression-like behavior has also been reported and even detected in an “early” disease state in genetically lupus-prone rodents, e.g., the MRL/lpr (4), NZB × NZW F1, and 564Igi strains (5). Recent advances point to an immune-mediated neuroinflammatory pathogenesis (6, 7), but the detailed mechanism underlying SLE-related depression has not been clarified. Furthermore, owing to the heterogeneity of depression in individuals with SLE, symptom-based diagnostic approaches are inadequate, and effective antidepressant treatments are lacking.

Correct synaptic wiring is a basic prerequisite for accurate synaptic connectivity and neuronal function. Increased elimination of synapses, resulting in an overall loss of synapses, is observed in several pathological conditions, including brain inflammation (8) and SLE (9). Microglia-mediated engulfment of synaptic processes, which is a potential mechanism underlying synapse loss, is increased in these diseases. Moreover, the number and activation of microglia are altered in the brains of individuals with SLE (10). Our previous work demonstrated that increased synaptic stripping by microglia accounts for early neuropsychiatric (NP) symptoms in lupus mice (9). However, the mechanism by which microglia recognize specific synapses for precise pruning remains unclear. Moreover, in SLE, which and how activated systemic immune factors affect synaptic pruning in the brain and whether this systemic signal can be targeted to prevent brain injury and alleviate depression in patients with SLE are unclear. Elucidating these problems would be helpful for early and precise intervention in patients with SLE-related depression.

In addition to microglia, cytotoxic T cells and systemic immune signals can also target neurons in inflammation-related conditions, including viral infections (11), autoimmune diseases (12), and neurodegenerative disorders (13). The cellular and molecular bases of T cell– and T cell–derived cytokine–driven synaptic pathology are currently largely unclear. A recent study proposed that neurons themselves function as intermediaries between CD8^+^ T cells and phagocytes, driving synaptic loss and motor coordination impairment in encephalitis (11). Additionally, phagocytes can be activated by immune factors (14) and act as effectors of T cell–driven brain damage (15). Thus, it can be hypothesized that microglia are responsible for systemic immune factor–driven synaptic degeneration, but this needs to be verified experimentally.

Recently, accumulating evidence has indicated that systemic mesenchymal stromal cell (MSC) transplantation (MSCT) ameliorates tissue damage in a variety of human diseases, such as graft-versus-host disease (GvHD), autoimmune encephalomyelitis, diabetes, liver fibrosis, Alzheimer disease, and other age-related degenerative diseases (16–21). Multiple mechanisms, including paracrine cytokine secretion (21), direct interplay with immune cells (22), extracellular vesicles (19, 20), and epigenetic regulation (23), have been identified as contributors to the therapeutic effect of MSCT. Our previous studies confirmed that MSCT has therapeutic effects on patients with SLE and SLE model mice (23–26). However, the extent to which MSCT ameliorates NP symptoms, particularly depression, and rescues synaptic structure impairment in patients with SLE remains largely unclear.

In this study, we applied MSCT to both *Fas*-deficient MRL/lpr mice and pristane-induced lupus mice to explore the early neuroprotective potential of MSCT. We show that MSCT rescues impaired synaptic density and alleviates depression-like behaviors in young (5- to 8-week-old) lupus mice by reducing systemic T helper 1 (Th1) cell–derived IFN-γ levels to inhibit neuronal JAK/STAT1 signaling and downstream CCL8 expression and then inhibit neuron-coordinated synapse elimination by microglia in the brain. Our data provide insights into the molecular mechanisms underlying immune-mediated psychopathy and suggest that biotherapies, such as MSCT, represent potential treatments and require clinical attention.

## Results

### Lupus mice develop increased depression-like behavior in the early stage of SLE.

**Figure 1:**
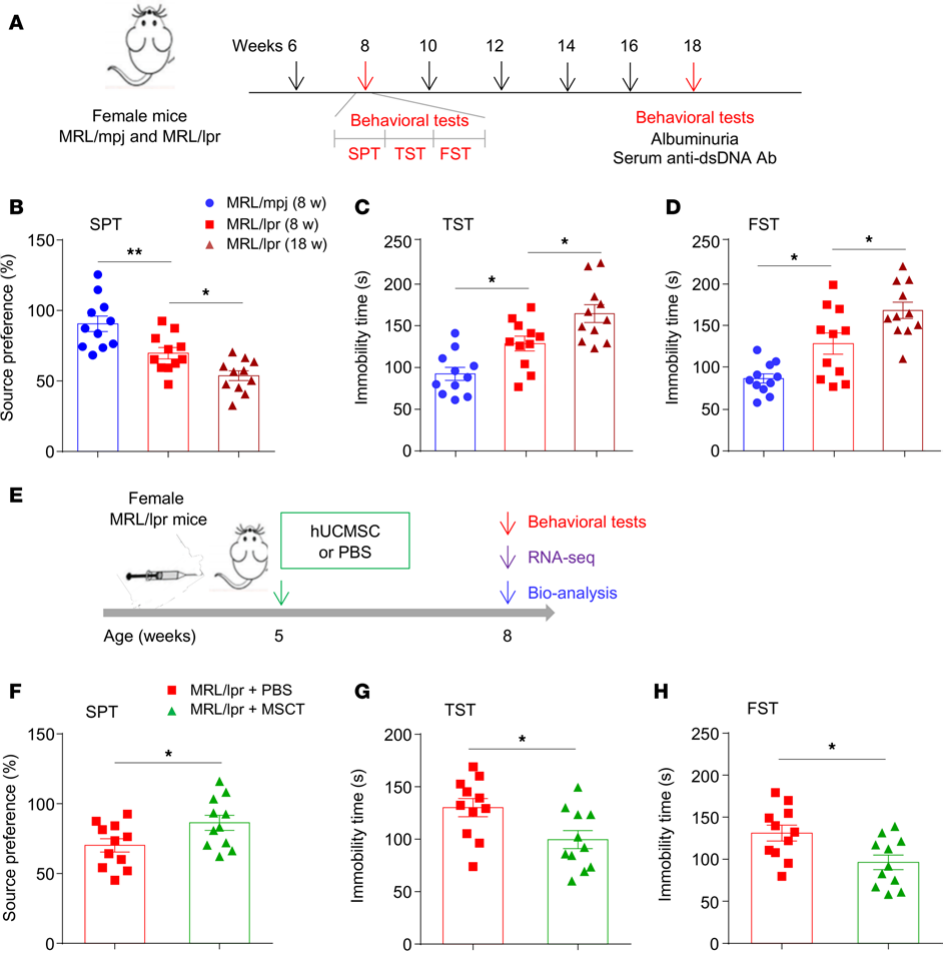
MSCT alleviates depression in MRL/lpr mice. (**A**) Timeline of the experimental procedure for MRL/lpr mice. (**B**–**D**) Evaluation of depression-like behavior in MRL/lpr mice and wild-type (MRL/mpj) controls (*n* = 11 mice/group). The SPT (**B**), TST (**C**), and FST (**D**) were performed at 8 and 18 weeks. (**E**) Experimental protocol for the treatment of MRL/lpr mice by MSCT. (**F**–**H**) Effects of MSCT on depression-like behavior in MRL/lpr mice (*n* = 11 mice/group). Five-week-old MRL/lpr mice were intravenously injected with hUCMSCs (5 × 10^5^ cells in 500 μL of PBS) or PBS (as a control). The SPT (**F**), TST (**G**), and FST (**H**) were performed 3 weeks after MSC/PBS injection. The data are presented as mean ± SEM. *P < 0.05; **P < 0.01, determined using 1-way ANOVA followed by Tukey’s post hoc test (**B**–**D**), or 2-tailed unpaired *t* test (**F**–**H**). SPT, sucrose preference test; TST, tail suspension test; FST, forced swim test. See also Supplemental Figures 1–4.

To investigate the relationship between SLE and depression, we monitored NP changes in MRL/lpr mice, a well-established lupus-prone murine model (27). MRL/lpr mice developed typical SLE-related pathological changes, as evidenced by the presence of anti–double-stranded DNA (anti-dsDNA) antibodies in the serum beginning at 8 weeks, the occurrence of albuminuria, and increased spleen size and weight at 18 weeks (Supplemental Figure 1, A–D; supplemental material available online with this article; https://doi.org/10.1172/jci.insight.181885DS1). To measure depression-like behavior, we first performed the sucrose preference test (SPT) to evaluate the tendency of the mice to consume sucrose at 8 weeks (early stage) and 18 weeks (active stage) (Figure 1A). Compared with control mice, MRL/lpr mice presented a decreased sucrose preference at 8 weeks of age, which was aggravated at 18 weeks of age (Figure 1B). Two other tests (the tail suspension test [TST] and forced swim test [FST]) were employed. The immobility time of MRL/lpr mice was markedly higher than that of control mice in both the TST (Figure 1C) and the FST (Figure 1D) at 8 weeks, and this depressive-like behavior was aggravated at 18 weeks of age. As reduced activity may contribute to immobility in the TST and FST, we then investigated locomotor activity with the open field test (OFT) and found that MRL/lpr mice presented no general locomotor defects (Supplemental Figure 1, E and F).

For confirmation, we evaluated the behavior of C57BL/6J mice in which lupus was induced by intraperitoneal (i.p.) injection of pristane (28). Ten weeks after pristane injection (early stage), the mice were subjected to behavioral tests (Supplemental Figure 2A). Compared with mineral oil–treated control mice, pristane-treated mice presented a reduced sucrose preference and increased immobility time in both the TST and the FST (Supplemental Figure 2, B–D) and developed typical lupus nephritis–like changes, with increased albuminuria levels and C3 deposition until 5–6 months after pristane treatment (active stage; Supplemental Figure 2, E and F), as reported previously (28). Together, these data indicate that both genetically prone and induced-lupus mice develop depression in the early stage of the disease before the appearance of overt peripheral pathology.

### MSCT alleviates depression by attenuating dendritic loss in the brains of lupus mice.

MSCT can alleviate various CNS disorders, including autoimmune-related and nonautoimmune-related disorders (21, 29). Here, we investigated whether the transplantation of human umbilical cord MSCs (hUCMSCs), which were delineated (Supplemental Figure 3, A and B) and prepared as previously reported (30), alleviated depression-like behavior in SLE models. We treated 5-week-old MRL/lpr mice with hUCMSCs and assessed depressive behavior 3 weeks later (Figure 1E). Compared with PBS, MSCT ameliorated the decrease in sucrose preference in MRL/lpr mice (Figure 1F). The decrease in immobility time in both the TST (Figure 1G) and the FST (Figure 1H) was markedly ameliorated in the mice that underwent MSCT. In addition, we conducted complementary experiments in pristane-induced lupus mice. Four weeks after pristane injection, the mice were infused with MSCs or PBS and subjected to behavioral tests 6 weeks later (Supplemental Figure 4A). The results observed in the pristane-induced model were consistent with those in MRL/lpr mice (Supplemental Figure 4, B–D).

**Figure 2:**
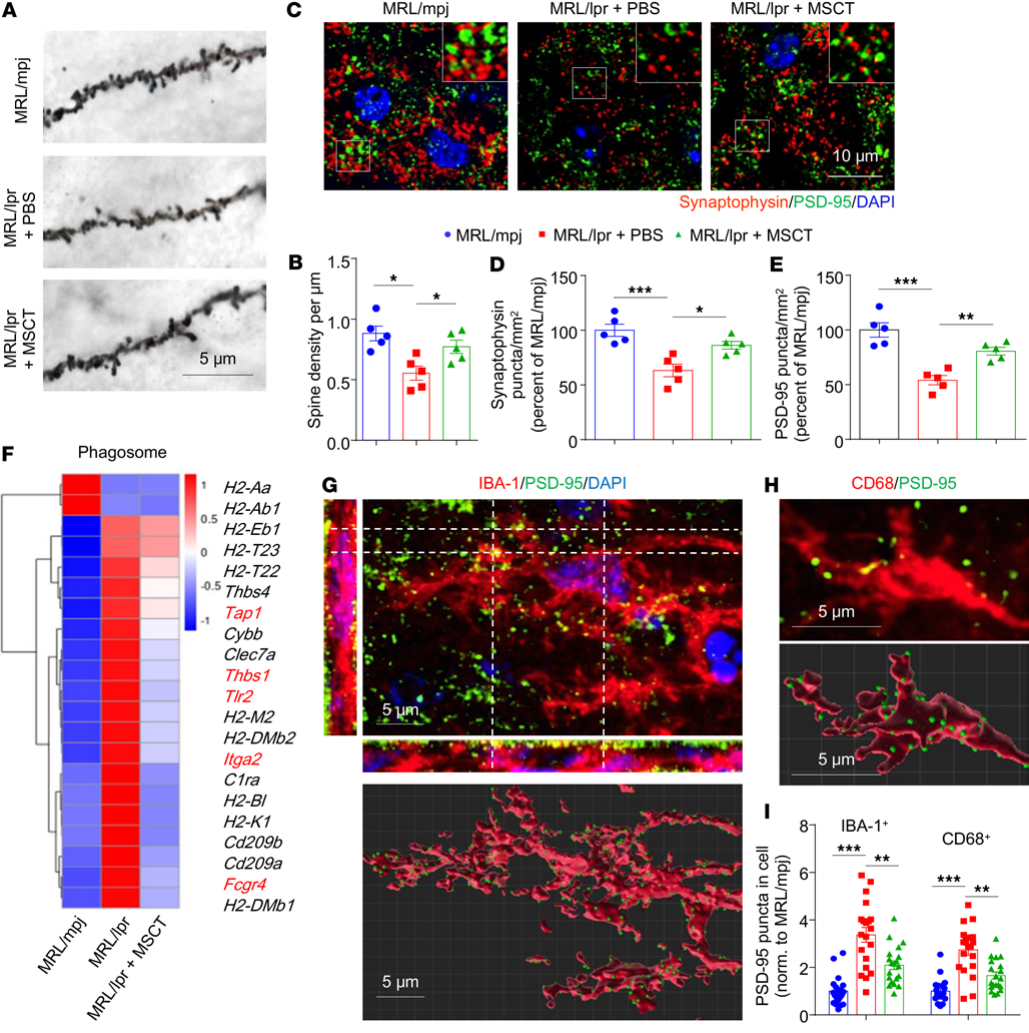
MSCT inhibits microglia-mediated synaptic stripping to rescue dendritic loss in the brains of lupus mice. (**A** and **B**) Images and quantification of Golgi-stained dendritic spines in dentate gyrus granule neurons from MRL/mpj mice and MSCT/PBS-treated MRL/lpr mice (*n* = 5 mice/group). Scale bar: 5 μm. (**C**–**E**) Immunostaining of presynaptic (synaptophysin, red) and postsynaptic (PSD-95, green) proteins in hippocampal sections from MRL/mpj mice and MSCT/PBS-treated MRL/lpr mice (**C**) and quantification of the levels of these proteins (**D** and **E**) (*n* = 5 mice/group, with an average of 3–4 slices per mouse). Scale bar: 10 μm. (**F**) Heatmap showing the relative expression of significantly altered genes in the hippocampi of MRL/mpj and MRL/lpr mice treated with or without MSCT according to RNA-seq (see Methods). The color scale represents the genewise *z* score calculated from the normalized gene expression levels. Each column represents an individual group (*n* = 3 mice/group). (**G** and **H**) Orthogonal view of a microglial cell (IBA-1, red) showing PSD-95 inclusions (green, upper in **G**) and colocalization with CD68 (red, upper in **H**). These findings suggest that microglia eliminate synapses in the hippocampi of MRL/lpr mice. Bottom: 3D reconstruction of the cell in **G** and lysosome in **H** (red) and PSD-95 inclusions (green) with Imaris software. Scale bars: 5 μm. (**I**) Quantification of reconstructed PSD-95^+^ spheres and PSD-95^+^CD68^+^ spheres inside microglia in the indicated mice (*n* = 20 cells from 4–5 mice). The data are presented as mean ± SEM. *P < 0.05; **P < 0.01; ***P < 0.001 by 1-way ANOVA followed by Tukey’s post hoc test. See also Supplemental Figures 5 and 6.

To determine whether MSCT altered key pathological deficits associated with depressive behavior, we measured the numbers of neurons and dendritic spines in the brains of lupus mice. MRL/lpr mice showed no anatomical damage (Supplemental Figure 5A), no change in the ratio of brain weight to body mass (Supplemental Figure 5, B and C), and no marked neuronal loss in the hippocampus at 8 weeks of age (Supplemental Figure 5, D and E). Golgi staining of hippocampal sections revealed that the number of dendritic spines was significantly reduced in MRL/lpr mice and that this decrease was effectively prevented by MSCT (Figure 2, A and B). In parallel, superresolution fluorescence microscopy revealed that the numbers of both presynaptic (synaptophysin, SYP) and postsynaptic terminals (PSD-95) were substantially decreased in the brains of MRL/lpr mice and that this change was reversed by MSCT (Figure 2, C–E). MSCT also alleviated early-onset splenomegaly and reduced serum anti-dsDNA antibody levels (Supplemental Figure 5, F and G). MSCT-mediated preservation of synaptic terminals was also observed in the pristane-induced model (Supplemental Figure 5H). These results indicate that MSCT substantially alleviates depression-like behavior and prevents hippocampal dendritic loss in lupus mice.

### MSCT inhibits microglia-mediated synaptic stripping to rescue dendritic loss.

Microglia act as key mediators of brain circuit connectivity by pruning synapses (31). We then hypothesized that MSCT rescued depression-like behavior and synapse loss in MRL/lpr mice by preventing microglia-mediated synaptic elimination. To test this hypothesis, we employed RNA sequencing (RNA-seq) to compare the gene transcription profiles of microglia isolated from mice after MSCT. The purity of the microglia isolated with an anti-CD11b microbead kit was verified by flow cytometry (Supplemental Figure 6A). Gene Ontology enrichment analysis confirmed that genes enriched in reactive microglia-related pathways, including phagocytosis, engulfment, and the cellular response to IFN-γ and the NF-κB signaling pathway, were upregulated in sorted microglia from MRL/lpr mice (Supplemental Figure 6, B and C). Notably, the expression of phagocytosis-related genes was also upregulated in the hippocampi of MRL/lpr mice, and the changes in the expression of most of these genes were reversed by MSCT (Figure 2F). Immunostaining analysis revealed that MSCT reversed the increase in the phagocytic activity of monocytes in MRL/lpr mouse brain, as evidenced by a reduction in the number of CD68^+^IBA-1^+^ microglia and the staining intensity of CD68 in the hippocampus (Supplemental Figure 6, D–F). Moreover, almost all of the IBA-1^+^ cells in the CNS of MRL/lpr were enriched with the microglia-specific marker TMEM119 (32) (Supplemental Figure 6G), which confirmed the microglia-derived origin of these cells.

We further performed staining for presynaptic (SYP), postsynaptic (PSD-95), microglial (IBA-1), and lysosomal (CD68) markers in mouse hippocampal slices and then evaluated synapse phagocytosis via 3-dimensional (3D) reconstruction to confirm synapse engulfment by microglia (Figure 2, G and H). Quantitative analysis revealed that the number of PSD-95^+^ puncta in microglia (both within and outside the CD68^+^ lysosomal compartment) in MRL/lpr mice was markedly higher than that in control mice and that this change was reversed by MSCT (Figure 2I). We also detected an increase in the number of PSD-95^+^ puncta in IBA-1^+^ cells in pristane-induced lupus mice, which was alleviated by MSCT (Supplemental Figure 5, I and J). In addition, depletion of microglia with PLX5622, a selective inhibitor of colony-stimulating factor 1 receptor (CSF1R), maintained dendritic integrity and alleviated depressive behavior in MRL/lpr mice (Supplemental Figure 6, H–K), suggesting that microglia-mediated engulfment contributes to synapse loss in lupus mice. These results collectively indicate that MSCT ameliorates depression in a lupus mouse model by inhibiting microglial phagocytosis and rescuing synaptic connectivity.

### MSCT targets neuronal CCL8 signaling to inhibit microglial apposition and synaptic stripping.

**Figure 3:**
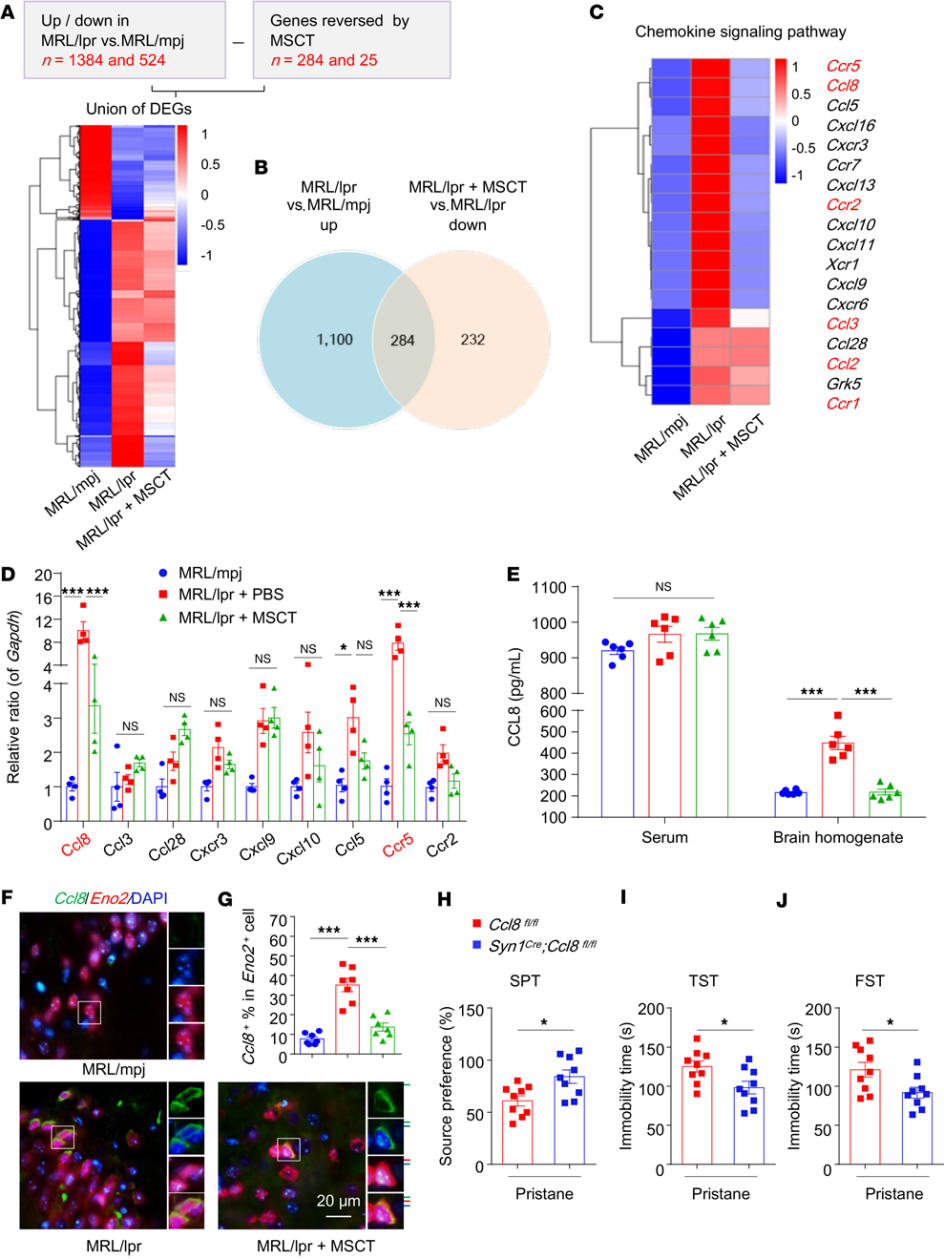
MSCT inhibits microglial apposition and synaptic stripping by targeting neuronal CCL8 signaling. (**A** and **B**) Mouse hippocampal tissues were harvested, and RNA-seq was subsequently performed. The heatmap shows all genes whose expression was altered in MRL/lpr mice compared with MRL/mpj mice and whose expression was further reversed by MSCT (**A**). The Venn diagram (**B**) shows overlapping genes that were significantly upregulated in MRL/lpr mice versus controls and whose expression was reversed by MSCT (downregulated in MSCT-treated mice versus MRL/lpr mice). (**C**) Heatmap showing the significantly altered genes enriched in “chemokine signaling pathway.” Each column represents an individual group (*n* = 3 mice/group). The color scale represents the genewise *z* score calculated from the normalized gene expression levels. Genes are ordered by hierarchical clustering (**A** and **C**). (**D**) Validation of selected genes in a unique set of mice by qPCR (*n* = 4 mice/group). (**E**) Validation of CCL8 levels in the serum and hippocampal homogenates of each group by ELISA (*n* = 6 mice/group). (**F**) Representative images of FISH for *Ccl8* (green) and the neuronal marker *Eno2* (red) in hippocampal sections from the indicated mice. Scale bar: 20 μm. (**G**) Quantitative results for *Ccl8*^+^ neurons in the hippocampal sections (*n* = 7 mice/group). (**H**–**J**) Evaluation of depression-like behavior in pristane-treated *Ccl8^fl/fl^* and *Syn1^Cre^*;*Ccl8^fl/fl^* mice (*n* = 9 mice/group). Ten weeks after pristane injection, the mice were subjected to the SPT (**H**), TST (**I**), and FST (**J**). The data are presented as mean ± SEM. *P < 0.05; ***P < 0.001, determined using 1-way ANOVA followed by Tukey’s post hoc test (**D** and **E**), or 2-tailed unpaired *t* test (**H**–**J**). NS, not significant; DEGs, differentially expressed genes; SPT, sucrose preference test; TST, tail suspension test; FST, forced swim test. See also Supplemental Figures 7 and 8.

To further determine the mechanism underlying microglial apposition and synapse stripping, we collected hippocampal tissue from MRL/mpj, MRL/lpr, and MRL/lpr + MSCT mice and performed RNA-seq. Among the 1,908 differentially expressed transcripts (1,384 upregulated, 524 downregulated; cutoff of a 1.5-fold change; *P* < 0.05) in the hippocampi of MRL/lpr mice, MSCT reversed the changes in the expression of 309 transcripts (284 downregulated, 25 upregulated in the MRL/lpr + MSCT group) (Figure 3, A and B, and Supplemental Figure 7A). The top differentially expressed and revised genes were involved in the “cytokine-cytokine receptor interaction” and “chemokine signaling pathway” (Supplemental Figure 7B). Neurons actively regulate the recruitment of phagocytes via paracrine mechanisms (11, 33). We then hypothesized that there are soluble factor(s) produced by neurons that might be responsible for phagocytic microglial apposition. Next, we screened genes from the RNA-seq data and published literature that might be involved in this process and identified CCL8 as a candidate (Figure 3C). The results of quantitative PCR and ELISA confirmed that the expression of CCL8 in the hippocampi of MRL/lpr mice was increased and that this increase was reversed by MSCT (Figure 3, D and E, and Supplemental Figure 7C). The CC-motif chemokine receptor type 5 (*Ccr5*), which interacts with CC-motif chemokine ligand 8 (*Ccl8*), was highly expressed in the lupus brain and suppressed by MSCT, as revealed by the RNA-seq data and confirmed by PCR (Figure 3, C and D). RNAscope in situ hybridization (FISH) revealed elevated *Ccl8* mRNA expression in MRL/lpr mouse brains, which was detected mainly in *Eno2*^+^ neurons but not in TMEM119^+^ microglia (Figure 3, F and G, and Supplemental Figure 7D). This finding is consistent with the increase in the number of neurons contacted by IBA-1^+^ microglia in the brains of lupus mice (Supplemental Figure 8, I and J). Additionally, MSCT reduced the number of *Ccl8*^+^ neurons in the brains of lupus mice (Figure 3, F and G).

To examine directly whether neuronal CCL8 chemokine signaling is required for microglial chemotaxis and synaptic injury in SLE, we used *Syn1^Cre^;Ccl8^fl/fl^* mice in which *Ccl8* was conditionally knocked out in neurons in the brain parenchyma. Both *Ccl8*-knockout mice and their controls (*Ccl8^fl/fl^*) were subjected to pristane or mineral oil injection (Supplemental Figure 8A). Compared with age-matched littermate controls (*Ccl8^fl/fl^*), *Syn1^Cre^;Ccl8^fl/fl^* mice were born normally and presented no obvious abnormalities in body weight or locomotor performance (Supplemental Figure 8, B–D). Neurons expressing *Ccl8* were detected in the brains of pristane-treated *Ccl8^fl/fl^* but not *Syn1^Cre^;Ccl8^fl/fl^* mice (Supplemental Figure 8E), confirming the conditional neuronal deletion of *Ccl8* in these mice. Further analysis revealed that neuronal *Ccl8* knockout largely protected mice from depression, as evidenced by reduced depressive behavior (Figure 3, H–J), an increase in the number of synapses in the CA1 region (Supplemental Figure 8, F–H), and a decrease in the number of microglia adjacent to neurons (Supplemental Figure 8, I–L), without affecting the phagocytic activity of microglia (Supplemental Figure 8M).

Together, these findings indicate that aberrant expression of neuronal CCL8 plays an important role in phagocyte apposition and that inhibition of CCL8 expression may be responsible for the ability of MSCT to prevent synapse elimination and alleviate depression in patients with SLE.

### IFN-γ and neuronal JAK/STAT1 signaling contribute to MSCT-mediated neuroprotection in MRL/lpr mice.

**Figure 4:**
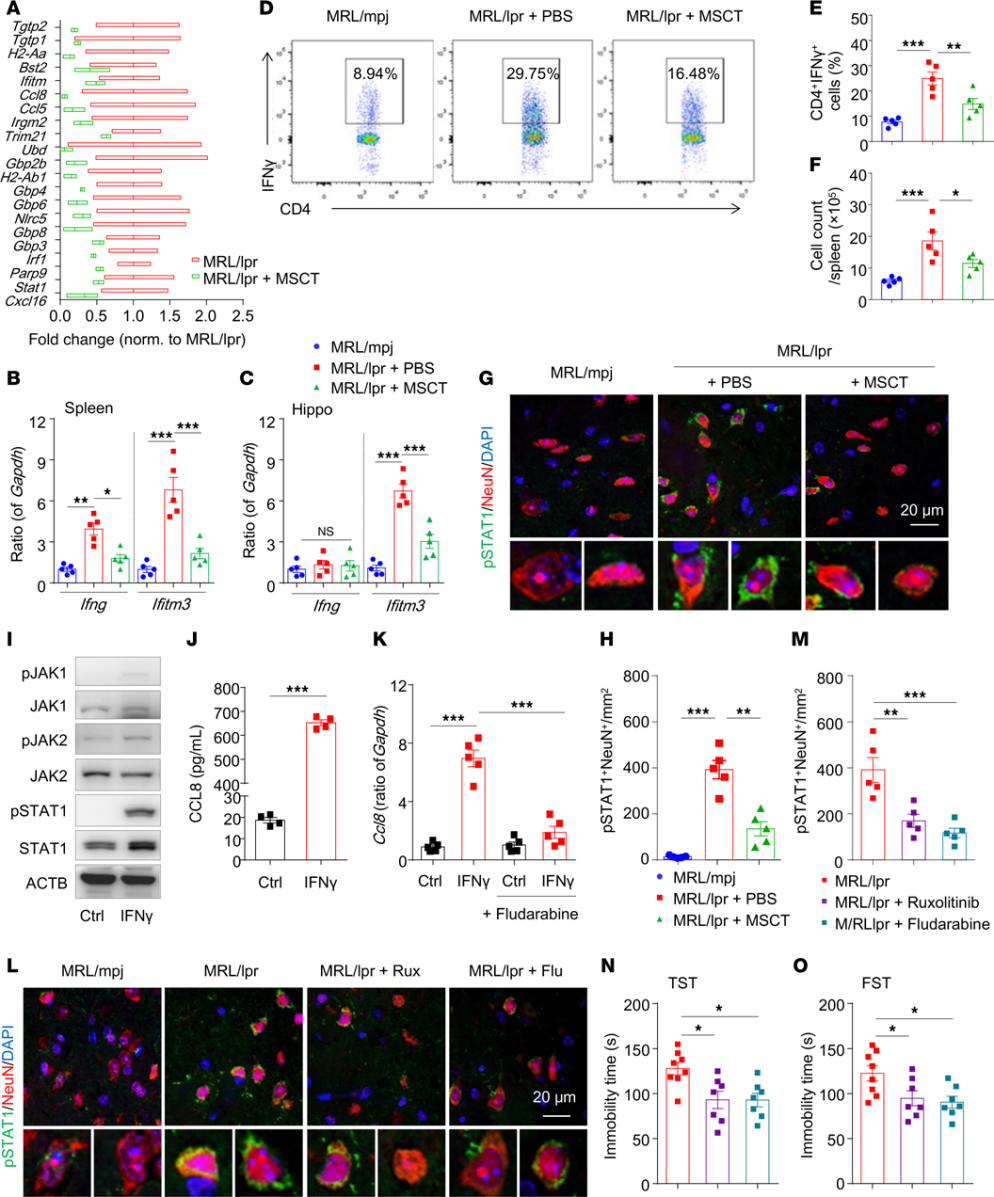
IFN-γ/JAK/STAT1 signaling contributes to MSCT-mediated improvements in neuronal function in recipient MRL/lpr mice. (**A**) The expression of IFN response genes was significantly altered by MSCT in the hippocampi of MRL/lpr mice (*n* = 3 mice/group). (**B** and **C**) qPCR analysis of *Ifng* and *Ifitm3* expression in the spleens and hippocampi of the indicated mice (*n* = 5 mice/group). (**D**–**F**) Representative flow cytometric analysis of splenic CD4^+^IFN-γ^+^ (Th1) cells in the mice. The percentages (**E**) and numbers (**F**) of Th1 cells are shown in the right 2 panels (*n* = 5 mice/group). (**G** and **H**) Representative images of brain sections coimmunostained for p-STAT1 together with a neuronal marker (NeuN^+^) and quantification of positive cells in the CA1 region of the indicated groups (*n* = 5 mice/group). Scale bar: 20 μm. (**I**–**K**) IFN-γ (10 ng/mL) was used to treat hippocampal neurons. (**I**) p-JAK1, p-JAK2, and p-STAT1 levels were measured by Western blotting 30 minutes after treatment. (**J**) The expression of CCL8 was measured by ELISA 24 hours after treatment. (**K**) *Ccl8* expression was assessed by RT-PCR 6 hours after treatment with IFN-γ and fludarabine. (**L**–**O**) Pharmacological inhibition of JAK1/2 or STAT1 in MRL/lpr mice. Ruxolitinib (Rux, p.o.) or fludarabine (Flu, i.p.) was given to MRL/lpr mice from 5 to 8 weeks of age. Immunostaining (**L**) and quantification (**M**) of p-STAT1^+^NeuN^+^ cells in the hippocampal sections were performed 3 weeks after pharmacological inhibitor treatment (*n* = 5 mice/group). Scale bar: 20 μm. The TST (**N**) and FST (**O**) were performed at the end of the experiment (*n* = 7–8 mice/group). The data are presented as mean ± SEM. *P < 0.05; **P < 0.01; ***P < 0.001, determined using 1-way ANOVA followed by Tukey’s post hoc test (**B**, **C**, **E**, **F**, **H**, **K**, **M** and **N**) or Dunnett’s multiple comparisons test (**O**), or 2-tailed unpaired *t* test (**J**). Ctrl, control; TST, tail suspension test; FST, forced swim test. See also Supplemental Figure 9.

Because neurons in the healthy brain express low levels of CCL8 and microglia are not highly active, we wondered which factors in the brain are involved in the pathology of lupus-related depression. Elevation of the Th1 cytokine IFN-γ is a hallmark of SLE (34), and increased IFN-γ levels in the brain can therefore affect both neurons and microglia. IFN-γ receptor (IFNγR) activation triggers JAK1/2-dependent phosphorylation of STAT1, which then homodimerizes and translocates to the nucleus, where it stimulates the transcription of genes containing IFN-γ–activated sequence (GAS) elements (35) (Supplemental Figure 9A). By examining RNA-seq reads, we found that genes enriched in both the “JAK-STAT signaling pathway” and “response to interferon-gamma pathway” were markedly upregulated in the hippocampi of MRL/lpr mice (Supplemental Figure 9B), which is consistent with the upregulation of genes enriched in “response to interferon-gamma pathway” in isolated microglia from MRL/lpr mice (Supplemental Figure 6, A and B). The upregulation of IFN-γ–responsive genes in the hippocampal microglia of MRL/lpr mice (Supplemental Figure 9C) was reversed by MSCT (Figure 4A). A subsequent qPCR analysis revealed that *Ifng* and *Ifitm3*, which are IFN-γ–stimulated genes, were upregulated in the spleens of MRL/lpr mice and that the expression of both genes was reversed by MSCT (Figure 4B). *Iftm3* but not *Ifng* was upregulated in the hippocampi of MRL/lpr mice (Figure 4C), suggesting that systemic IFN-γ activated IFNγR signaling in the brain and that MSCT might inhibit brain IFNγR signaling by targeting systemic IFN-γ. In lupus, CD4^+^IFN-γ^+^ T cells (Th1 cells), the number of which is increased, are the major sources of systemic IFN-γ. Indeed, flow cytometric analysis revealed that MSCT reduced the frequency and number of CD4^+^IFN-γ^+^ T cells, but not CD8^+^IFN-γ^+^ T cells, in the spleens of MRL/lpr mice (Figure 4, D–F, and Supplemental Figure 9, D–F). Coimmunostaining analysis revealed that the number of phosphorylated STAT1^+^ (p-STAT1^+^) cells, which were colocalized with mainly NeuN^+^ neurons, was increased in the hippocampi of MRL/lpr mice, and that this change was reversed by MSCT (Figure 4, G and H, and Supplemental Figure 9, H–J). To further confirm the involvement of JAK/STAT1 in neuronal CCL8 secretion, we treated primary mouse hippocampal neurons with IFN-γ in vitro. As shown in Figure 4K and Supplemental Figure 9G, IFN-γ was sufficient to upregulate *Ifitm3* and *Ccl8* expression. In addition, IFN-γ treatment increased the phosphorylation levels of JAK1, JAK2, and STAT1, and increased the secretion of CCL8 (Figure 4, I and J). Fludarabine (an inhibitor of STAT1) abrogated the increase in *Ccl8* expression in IFN-γ–treated neurons (Figure 4K). We further treated mice with ruxolitinib (an inhibitor of JAK1/2) or fludarabine (Supplemental Figure 9K) and found that both treatments substantially preserved the synaptic density (Supplemental Figure 9, L and M), reduced the number of p-STAT1^+^ neurons (Figure 4, L and M), and ameliorated depression in MRL/lpr mice (Figure 4, N and O). These results suggest that IFN-γ may increase the expression of CCL8 via key signal transduction molecules expressed in neurons, which promote microglial apposition to induce synaptic engulfment.

### Blockade of IFN-γ signaling rescues depressive behavior in MRL/lpr mice.

**Figure 5:**
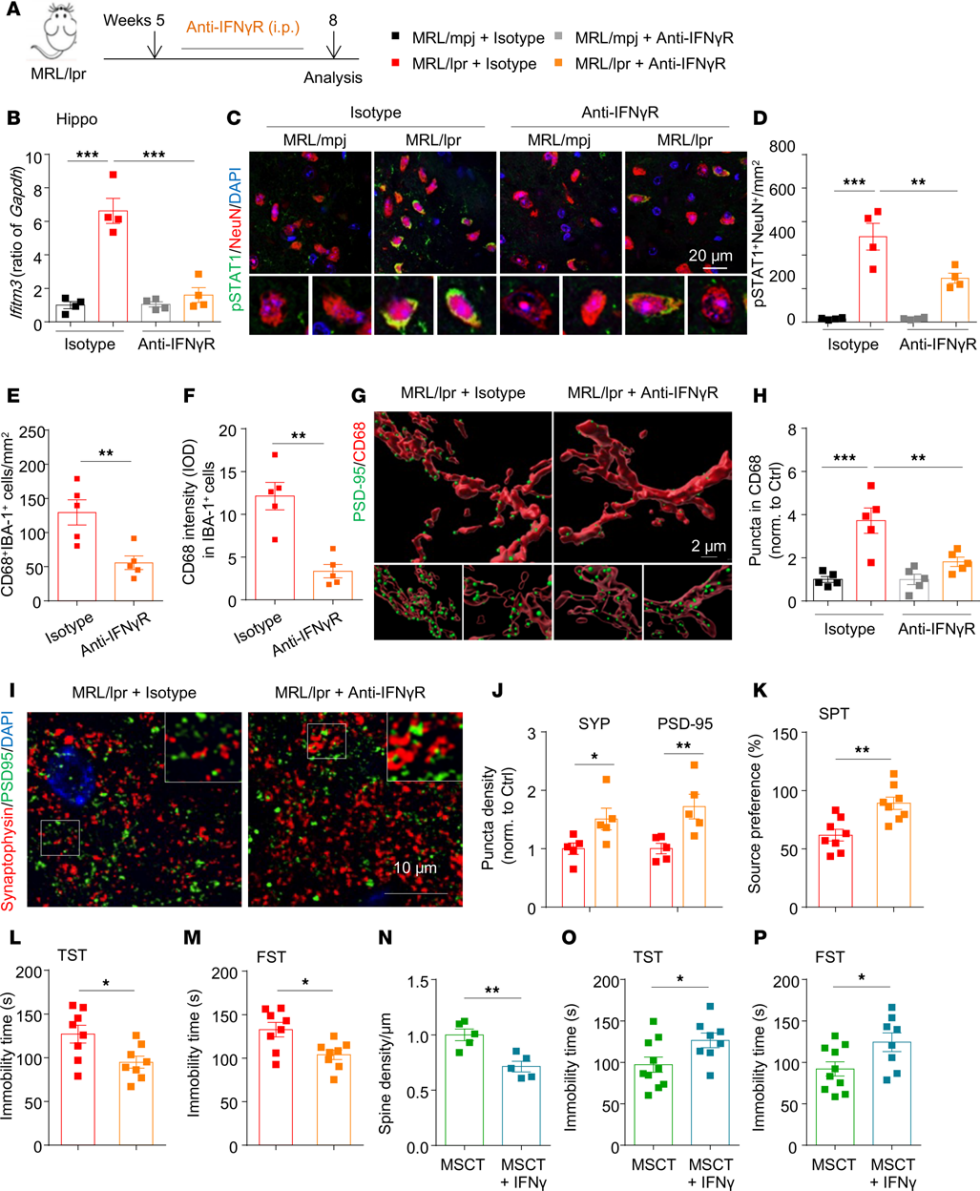
Blockage of IFN-γ signaling rescues depression phenotypes in MRL/lpr mice. (**A**) Experimental protocol for model establishment and analysis. (**B**) qPCR analysis of *Ifitm3* expression in the hippocampi of the indicated mice (*n* = 4 mice/group). (**C** and **D**) Representative images of brain sections coimmunostained for p-STAT1 together with a neuronal marker (NeuN^+^) and quantification of positive cells in the CA1 region of the indicated groups (*n* = 4 mice/group). Scale bar: 20 μm. (**E** and **F**) Quantification of IBA-1^+^CD68^+^ phagocytes (**E**) and CD68 staining intensity (**F**) in the hippocampi of the indicated groups (*n* = 5 mice/group). (**G** and **H**) Representative 3D reconstruction and quantification of engulfed PSD-95^+^ puncta (green) in CD68^+^ lysosomes (red) in the CA1 region of the indicated groups (*n* = 15–17 cells/group from 5 mice). Scale bar: 2 μm. (**I** and **J**) Immunostaining and quantification of presynaptic (SYP, red) and postsynaptic (PSD-95, green) proteins in hippocampal sections from the indicated mice (*n* = 5 mice/group, with an average of 3–4 slices per mouse). Scale bar: 10 μm. (**K**–**M**) The depression-like behavior of the indicated mice was assessed. The SPT (**K**), TST (**L**), and FST (**M**) were performed 3 weeks after antibody treatment (*n* = 8 mice/group). (**N**) Quantification of Golgi-stained dendritic spines of dentate gyrus granule neurons in MSCT (or MSCT plus IFN-γ–treated) MRL/lpr mice (*n* = 5 mice/group). (**O** and **P**) Depression-like behavior of the indicated mice was assessed via the TST (**O**) and the FST (**P**) (*n* = 8–10 mice/group). The data are presented as mean ± SEM. *P < 0.05; **P < 0.01; ***P < 0.001, determined using 1-way ANOVA followed by Tukey’s post hoc test (**B**, **D**, **H** and **J**), or 2-tailed unpaired t test (**E**, **F** and **K**–**P**). IOD, integrated optical density; SPT, sucrose preference test; TST, tail suspension test; FST, forced swim test. See also Supplemental Figure 10.

Our findings indicate that targeting IFN-γ signaling may be a key contributor to the antidepressant effect of MSCT in lupus mice. We then evaluated whether pharmacological interference with IFN-γ offers therapeutic benefits for depression in SLE patients. To this end, an IFN-γ signaling–blocking antibody (anti-IFNγR) or isotype control was injected into MRL/lpr mice twice a week for up to 3 weeks (Figure 5A). Seventy-two hours after the last injection, qPCR was performed. The results confirmed that IFN-γ–induced expression of the *Ifitm3* gene in the hippocampus and spleen was reduced in anti-IFNγR antibody–treated mice (Figure 5B and Supplemental Figure 10A). Histologic analysis revealed that the number of p-STAT1^+^ neurons was reduced (Figure 5, C and D) and that the number and phagocytic activity of IBA-1^+^ microglia were decreased in the hippocampi of anti-IFNγR antibody–treated mice (Figure 5, E and F). Moreover, microglial synaptic engulfment was markedly inhibited by anti-IFNγR antibody treatment, as analyzed by immunofluorescence and surface rendering analysis, which revealed a reduction in the number of PSD-95^+^ puncta in microglial phagosomes (Figure 5, G and H). Compared with isotype control treatment, anti-IFNγR antibody treatment substantially prevented synaptic loss (Figure 5, I and J) and ameliorated depressive behavior (Figure 5, K–M).

In a complementary approach, we assessed whether the administration of additional IFN-γ can induce or further potentiate the depressive phenotype and abrogate the antidepressant effect of MSCT in lupus. We found that IFN-γ–treated C57BL/6J mice presented a reduced sucrose preference, an increased number of IBA-1^+^ phagocytes, and impaired synaptic density in the hippocampus (Supplemental Figure 10, B–D). Also, compared with PBS-treated control mice, IFN-γ–treated MRL/lpr mice displayed more severe depression-like behavior and an increased number of IBA-1^+^ phagocytes in the brain (Supplemental Figure 10, E–G). Furthermore, IFN-γ treatment blocked the protective effect of MSCT on depression and its ability to preserve synaptic density in lupus mice (Figure 5, N–P, and Supplemental Figure 10, H and I).

### Patients with NPSLE show increased activation of the IFN-γ/JAK/STAT pathway and elevated CSF CCL8 levels.

**Figure 6:**
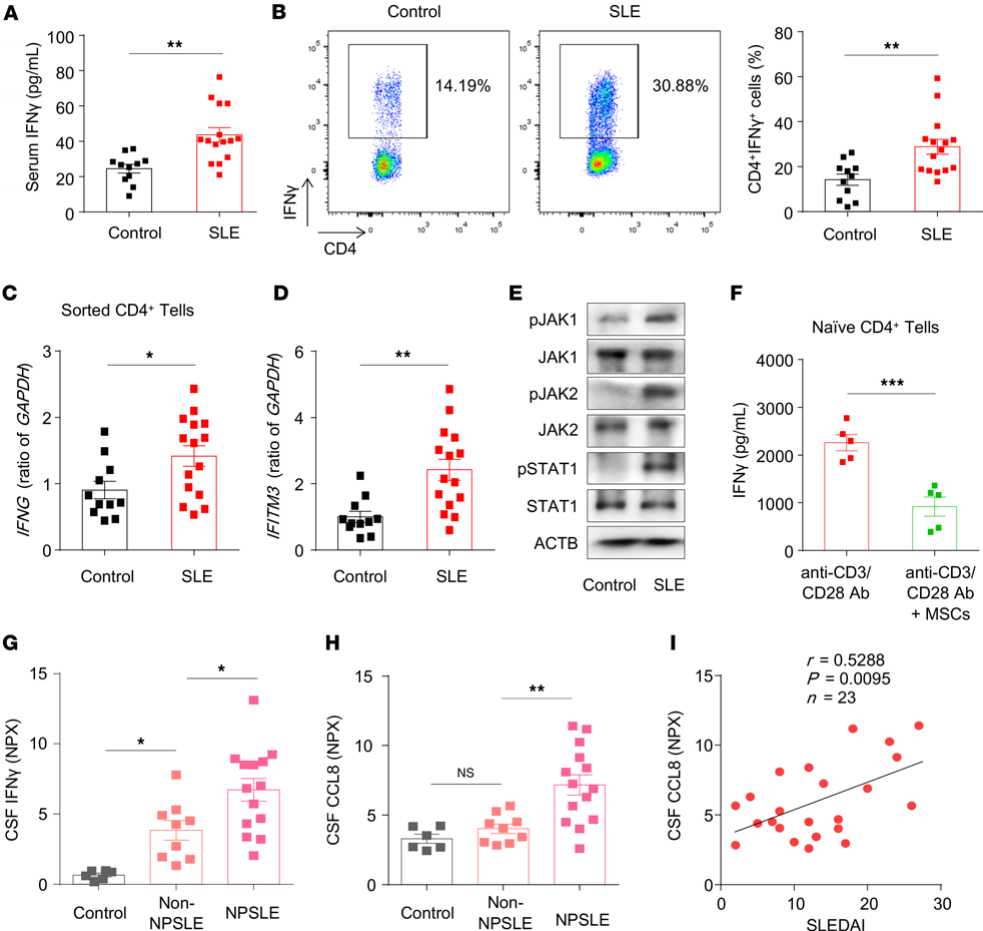
Patients with SLE showed activation of the IFN-γ/JAK/STAT pathway and elevated CCL8 levels in CSF. (**A**) ELISA revealed that patients with SLE (*n* = 15) had higher serum levels of IFN-γ than normal controls did (*n* = 11). (**B**) Flow cytometric analysis revealed that the number of CD4^+^IFN-γ^+^ cells in the peripheral blood was greater in patients with SLE (*n* = 15) than in normal controls (*n* = 11). (**C** and **D**) qPCR analysis showing the levels of *IFNG* and *IFITM3* in sorted CD4^+^ T cells from patients with SLE (*n* = 15) and normal controls (*n* = 11). (**E**) Western blot analysis revealed that p-JAK1, p-JAK2, and p-STAT1 levels were increased in CD4^+^ T cells from patients with SLE. (**F**) Protein levels of IFN-γ in the cultured supernatants of CD4^+^ T cells cocultured with/without MSCs under Th1-polarizing conditions for 5 days (*n* = 5). (**G** and **H**) IFN-γ and CCL8 levels in CSF samples from controls (*n* = 6), non-NPSLE patients (*n* = 9), and NPSLE patients (*n* = 14). (**I**) Analysis of the correlation between CCL8 levels in the CSF and SLEDAI scores in patients with SLE (*n* = 23). The data are presented as mean ± SEM. *P < 0.05; **P < 0.01; ***P < 0.001, determined using 2-tailed unpaired *t* test (**A**-**D** and **F**), or 1-way ANOVA followed by Sidak’s post hoc test (**G** and **H**). NS, not significant. The correlation coefficient *r* and *P* values were calculated via Spearman’s *r* test in **I**. SLEDAI, System Lupus Erythematosus Disease Activity Index. See also Supplemental Figure 11.

To confirm the relevance of our findings in mice to SLE patients, we conducted a pilot clinical study (Supplemental Table 5). ELISA analysis revealed that the serum levels of IFN-γ were markedly greater in patients with SLE than in control participants (Figure 6A). Flow cytometric analysis revealed that the percentage of CD4^+^IFN-γ^+^ cells in the circulation was markedly greater in patients with SLE than in control participants (Figure 6B). qPCR further confirmed that the levels of *IFNG* and the IFN-γ–induced gene *IFITM3* were elevated in sorted CD4^+^ T cells from patients with SLE (Figure 6, C and D). JAK/STAT1 signaling acts downstream of IFN-γ in the neurons, monocytes, and T cells of MRL/lpr mice (35). We found that the levels of p-JAK1, p-JAK2, and p-STAT1 were increased in sorted CD4^+^ T cells from patients with SLE (Figure 6E). Next, to determine the IFN-γ production inhibitory effect of MSCs, we cocultured MSCs with normal human naive CD4^+^ T cells via the Transwell system. ELISAs revealed that MSCs markedly reduced IFN-γ levels in the culture supernatants of T cells subjected to Th1 cell polarization (Figure 6F).

To investigate the mechanistic findings in the CNS, cerebrospinal fluid (CSF) samples from a group of 29 other individuals were used for a proximity extension assay with an inflammation panel of 92 predefined cytokines, chemokines, and other proteins (Supplemental Table 6). We found that 24 of the 54 specific proteins (detected in >70% of the samples), including IFN-γ, CCL8, IL-6, IL-8, and other inflammatory factors, were differentially expressed between SLE patients with NP symptoms (NPSLE) and those without NP symptoms (non-NPSLE) (*P* < 0.05, Supplemental Figure 11B). Among the markers, we confirmed that the level of IFN-γ was substantially increased in the CSF of patients with NPSLE (Figure 6G). The level of CCL8 in the CSF was significantly increased in patients with NPSLE but not in patients with non-NPSLE (Figure 6H). Furthermore, the CCL8 concentration in the CSF was positively correlated with the System Lupus Erythematosus Disease Activity Index (SLEDAI) score in patients with SLE (Figure 6I). Collectively, these data highlight that the IFN-γ/JAK/STAT1/CCL8 axis plays a role in microglia-mediated synaptic loss in lupus-related depression.

## Discussion

**Figure 7:**
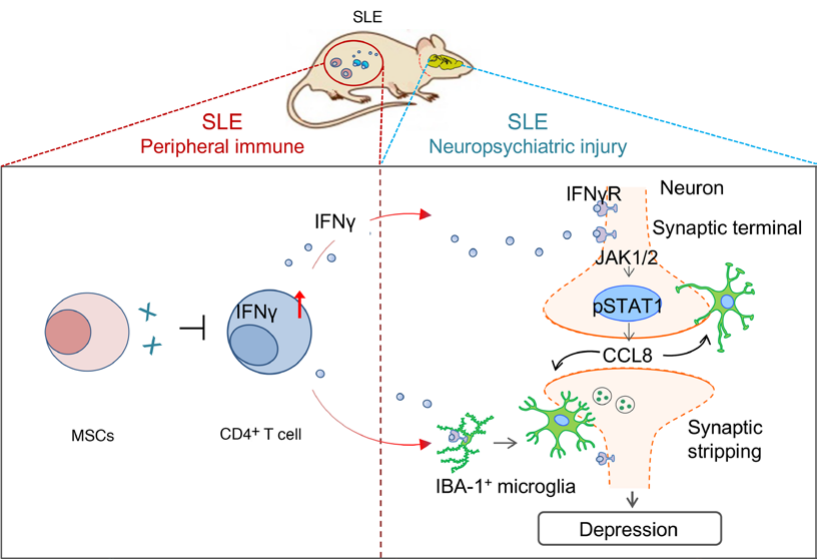
Graphical abstract. Schematic diagram of the mechanism by which MSCs inhibit neuron-coordinated synaptic stripping to alleviate depression in lupus mice by targeting IFN-γ/JAK/STAT1/CCL8 signaling.

Mood disorders, especially depression, are common and occur early in SLE and are specifically attributed to immune-initiated neuroinflammatory changes. Since NP events may initially be subtle when the blood-brain barrier (BBB) remains intact, developing non-BBB penetration requiring neuroprotective strategies, such as systemically targeted immunosuppressants, has important therapeutic implications. In the present study, we investigated the cellular and molecular mechanisms underlying depression in lupus and identified MSCT as an effective treatment strategy. We found that elevated systemic IFN-γ levels induce the expression of CCL8 in neurons and the activation of microglia, which contribute to synapse loss and depression-like behavior in individuals with lupus by increasing synaptic engulfment. We also revealed that transplanted hUCMSCs targeted the IFN-γ/CCL8 axis to alleviate microglial phagocytosis, synaptic defects, and depressive activity (Figure 7).

Since the first successful implementation of MSCT for the treatment of severe acute GvHD (18), MSCs from different sources (including bone marrow, adipose tissue, and umbilical cord) have been widely used for the treatment of various immune diseases in humans (16, 23, 36). Multiple mechanisms, such as cell-cell interactions, cytokine/chemokine secretion, and extracellular vesicles (EVs) delivery, have been proposed to contribute to the success of MSCT-based immune therapies (20, 22, 37). More recently, MSCT has been reported to have positive effects in treating CNS disorders (21, 38). Nevertheless, many factors, such as ethical issues, low yield, and the invasiveness of the procedures, limit the application of MSCT (21, 36). Owing to their advantages of high yield, ease of use, and fewer ethical problems, hUCMSCs represent a much better choice for clinical application. Allogenic hUCMSC-based therapies have shown beneficial effects in systemic sclerosis, Alzheimer disease, and other age-related degeneration models (16, 20, 21). These studies, combined with our recent publications (24, 25), suggest the efficacy and safety of hUCMSC-based therapies in treating SLE included autoimmune and nonautoimmune diseases. Psychiatric disorders, such as depression and anxiety, have been consistently linked to autoimmune-associated brain inflammation (3, 4, 6). However, whether hUCMSCs affect psychiatric symptoms in patients with autoimmune diseases and how to alleviate these symptoms remain unclear.

Recent evidence suggests that systemic factors are strong mediators of brain homeostasis, neurodegeneration, and psychiatric disorders (5, 13, 39). Depression is one of the most common affective disorders in patients with SLE and genetically lupus-prone mice (3, 4), and evidence suggests that immunological dysfunction in the brains of patients with SLE begins in the systemic circulation. Our previous works characterized the immunoregulatory effects of MSCT-based therapies in SLE (24, 30). Although evidence indicates that MSCT has therapeutic effects, little is known about the core factors that account for MSCT-mediated recovery of neuronal function (21). Therefore, clarifying whether and how MSCT exerts a neuroprotective effect from the perspective of systemic immune factors, especially in the presence of autoimmune-related CNS damage, is critical for both basic research and the clinical application of hUCMSCs in the treatment of inflammatory-associated CNS disorders.

In this study, we comprehensively evaluated the effects of MSCT on lupus-related depression. We demonstrated that the systemic Th1 cell cytokine IFN-γ plays an important role in the MSCT-mediated prevention of microglial synaptic elimination at least partially through inhibition of the IFN-γ/JAK/STAT1 signaling pathway. Our data supported the hypothesis that targeted regulation of immune signals is a promising strategy for the treatment of CNS diseases, which is consistent with previous reports (40, 41). Furthermore, as the safety and efficacy of MSCT have been suggested in many preclinical studies, we provide a rationale for the immediate application of MSCT in regulating systemic immune-brain communication and improving brain function in humans.

Recent studies have indicated that microglia, a macrophage-like resident brain cell population, are activated and express an “NPSLE-specific transcriptional signature,” which correlates with the severity of behavioral deficits in lupus mice (42). Sphingosine-1-phosphate (S1P) signaling (43), angiotensin-converting enzyme (ACE) (10), and NF-κB kinase subunit ε (44) seem to contribute to microglial activation, whereas complement components (10) and the IL-12/23p40 proteins (45) are downstream mediators of microglia-mediated neuronal damage in NPSLE. The activation of microglia results in the production of IL-6 and IL-18, which disturb hippocampal neurogenesis and mediate early diffuse NPSLE in the presence of an intact BBB prior to overt systemic disease (7). In addition, during sustained systemic inflammation, activated microglia phagocytose astrocytic endfeet and disrupt BBB permeability, initiating the leakage of systemic substances into the parenchyma and causing neuroinflammation (46). These findings suggest that the activation of microglia may be a key initiating event or at least an early event in NPSLE. It is conceivable that, in different pathological states, activated microglia potentially exacerbate neurological diseases via many mutually nonexclusive mechanisms. Impaired synaptic plasticity and altered dendritic spines have been observed as early-onset neurological deficits in lupus models (7, 9). Activated microglia eliminate dendritic spines and alter neuronal functions in the inflammatory brain (8). Here, we emphasized that the activation of brain-resident microglial phagocytosis contributes to the loss of dendritic complexity in early NPSLE, as the depletion of microglia by a CSF1R inhibitor preserved neuronal integrity and alleviated NPSLE. Thus, we also demonstrated what we believe is a novel mechanism underlying the neuroprotective effect of MSCT that involves microglia-mediated synaptic defects resulting from Th1 cell–neuron interactions. In addition to brain-resident microglia, blood-derived CD11b^+^ macrophages can also be pulled down by the microbead enrichment method we used, display engulfed synapses, and may contribute to the immunopathogenesis of encephalitis, but this was not directly addressed in our study. Furthermore, although we did not detect marked cellular infiltration in the brain parenchyma of the MRL/lpr strain at the early stage, when microglia were isolated for RNA-seq, the possibility that sorted CD11b^+^ cells were contaminated by macrophages cannot be completely excluded, as these cells are not being removed prior to sequencing.

Complement signaling has been suggested to contribute to microglia-mediated synapse removal (8, 31). However, it was not essential for microglial activation or MSCT-mediated disease prevention in the context of lupus-related depression (data not shown). Instead, by sequencing sorted cells from young lupus-prone mice, we identified an “IFNγ response” and “phagocytosis regulation” microglial signature. IFN-γ, a key Th1 cytokine, has been reported to play critical roles in regulating neuronal connectivity and social behavior (39). Pathologically, increased expression of IFN-γ–responsive genes along with phagocyte activation is observed in individuals with multiple sclerosis, virus infection, and HIV-associated neurocognitive disorders (11, 47, 48). IFN-γ is one of several cytokines detected in the serum and CSF of patients with SLE (34). Increased serum IFN-γ reaches the brain through the BBB and brain-CSF barrier (49), thereby affecting both neurons and microglia. We found that in the brains of lupus mice, IFN-γ–regulated genes are highly expressed, IFN-γ induces neurons to secrete CCL8 via JAK/STAT1 signaling, and microglia are activated and show increased phagocytosis following entry of IFN-γ into the brain. In vivo, lupus mice in which JAK/STAT1 or CCL8 signaling was inhibited were largely protected from depression and synapse loss. Thus, we speculated that in lupus, IFN-γ–induced neuronal expression of CCL8 represents a key step in communication between systemic immunity and microglia. CCL8 promoted the juxtaposition of active microglia to neurons to allow synaptic engulfment, in turn leading to synapse loss and depression. Collectively, our data support a model in which peripheral IFN-γ enters the brain to induce synapse loss and provide an explanation for the mechanism by which IFN-γ–stimulated neurons and microglia orchestrate synaptic engulfment. Furthermore, although the total number of synaptic inclusions in phagocytic microglia was reduced in anti-IFNγR–treated lupus mice, we cannot exclude the presence of other potential activators of microglial phagocytosis in lupus mice, as neuron-derived factors can also evoke phagocytosis via phagocytic receptors on microglia (50).

Moreover, the present data emphasize that peripheral Th1 cell–derived IFN-γ is a critical systemic contributor to early-onset neuronal damage in SLE. The percentage of Th1 cells among peripheral blood mononuclear cells (PBMCs) and IFN-γ levels in the serum are elevated in both lupus mice and patients with SLE (34, 51). Transplanted hUCMSCs targeted systemic Th1 cells and IFN-γ to alleviate depression. Accordingly, antibody-mediated neutralization of circulation IFN-γ ameliorated early-onset NPSLE through the inhibition of microglia-mediated synaptic stripping, and the neuroprotective effect of MSCT was blunted by systemic IFN-γ administration.

In addition to systemically derived cytokines, choroid plexus–infiltrated peripheral inflammatory cells have also been suggested as participants in NPSLE, especially at advanced (14 weeks old) (52) or late (16 weeks old) (53) stages of the disease. This raises the question of its involvement in early NPSLE in the presence of an intact BBB prior to overt systemic disease. We revealed that the MRL/lpr strain showed no marked cellular infiltration in the brain parenchyma before 5 weeks of age (9), which is in line with previous reports attributing an important role to CNS-resident cells and cytokines in the early development of NPSLE (7). However, at a later stage of the disease, when a breach in BBB integrity occurs, T and B cells can enter the brain, and aggravate neuronal damage (53, 54). Thus, the present study highlights that MSCT exerts an early neuroprotective effect in lupus by decreasing the number of systemic IFN-γ–producing CD4^+^ T (Th1) cells and reducing the entrance of IFN-γ into the brain. However, although Th1 cells infiltrating the brain parenchyma are not detected in young lupus mice, the potential infiltration of Th1 cells into the choroid plexus and MSCT prevents this from occurring in diseased lupus mice cannot be excluded. Moreover, while CNS lupus remains a heterogeneous disease with many probably causes and MSCT possesses many therapeutic targets, our findings suggest that some patients with NPSLE may benefit from MSCT in an IFN-γ–dependent manner in the early stage of the disease. Further studies are needed to clarify the effects and mechanisms of MSCT on different disease stages and different mental symptoms of NPSLE.

Collectively, our data demonstrate a link between lupus-related systemic immune activity, synaptic engulfment, and depression-like behavior, highlighting a role for neurons as intermediaries between T cells and microglia. We also provide evidence for the feasibility of MSCT in the treatment of lupus-related depression and other psychiatric disorders by targeting systemic immune signals.

## Methods

### Sex as a biological variable.

Our study exclusively examined female mice, because clinically, the incidence of SLE is substantially greater in females than in males. For human participants, both sexes were involved, with more females than males.

### Human participants.

SLE patients were recruited from the Department of Rheumatology and Immunology at Nanjing Drum Tower Hospital, the Clinical College of Nanjing Medical University, as we previously described (55). Age- and sex-matched healthy individuals or controls were enrolled at outpatient visits. Detailed information and sample collection information are provided in the Supplemental Methods.

### Mice.

MRL/MpJ-*Fas*^lpr^ (MRL/lpr) and MRL/MpJ (MRL/mpj) female mice were obtained from Shanghai Lingchang Biotechnology Corporation and genotyped according to protocols provided by The Jackson Laboratory for stocks 006825 and 000486. *Syn1^Cre^* mice [B6.Cg-Tg (Syn1-cre) 671Jxm/J; stock 003966] were purchased from The Jackson Laboratory. *Ccl8^fl/fl^* mice (no. CKOCMP-01361-Ccl8) were purchased from Cyagen Biosciences. For Cre-lox experiments, *Syn1^Cre^* mice were crossbred with *Ccl8^fl/fl^* mice. Female transgenic (*Syn1-cre^+/–^;Ccl8^fl/fl^*) animals aged 8 weeks were used. All animals were housed in the animal facility under standard laboratory conditions (12:12-hour light/dark cycle with free access to food and water).

### Treatment of mice and pharmacological intervention.

To establish the pristane-induced lupus mouse model, female C57BL/6J and *Ccl8* conditional knockout mice (8 weeks old) were given a single i.p. injection of 500 μL pristane (Sigma-Aldrich, P2870) or the same volume of mineral oil (Sigma-Aldrich, M5904) as a control. To block IFN-γ signaling in MRL/lpr mice, 5-week-old mice were given an i.p. injection of 150 μg of anti-IFNγR antibody (R&D Systems, MAB10262) or purified rat IgG2b isotype control antibody (R&D Systems) every 3.5 days for 3 weeks. To assess whether IFN-γ can induce or potentiate the depressive phenotype, both female C57BL/6J and MRL/lpr mice received i.p. injections of 10 ng of recombinant mouse IFN-γ protein (R&D Systems, 485-MI) or PBS, every 3.5 days for 2 weeks. MSC-treated MRL/lpr mice from another cohort received i.p. injections of 10 ng of IFN-γ protein or PBS every 3.5 days starting on day 7 after MSCT. MRL/lpr mice were randomly selected to receive oral gavage of ruxolitinib (30 mg/kg; Selleck, S1378) twice daily or i.p. injection of fludarabine (20 mg/kg; Selleck, S1491) once daily to inhibit JAK1/2 and STAT1 activity (11). For the microglia depletion experiments, 5-week-old MRL/lpr mice were treated with PLX5622 (1,200 mg/kg; MedChemExpress), which was incorporated into the AIN-76A rodent diet by Research Diets, Inc., for 3 weeks, as previously reported (56, 57).

### Tissue imaging and analysis.

For immunofluorescence analysis, the sections were scanned using a Zeiss LSM 710 confocal laser microscope. RGB color thresholds were used to identify positive staining. The mean fluorescence intensity in regions of interest (ROIs) was measured with ImageJ software (NIH). Synapse quantification and microglial engulfment analysis were performed as previously reported (9). For synapse quantification and stimulated emission depletion (STED) microscopy, hippocampal sections stained for SYP and PSD-95 were imaged using a Leica SP8 STED 3× microscope. The acquired images were deconvoluted via the Huygens package, and the number of pre- and postsynaptic puncta in each field was measured using ImageJ on the basis of the synaptic markers used. For microglial engulfment analysis, the sections were imaged using an Olympus SpinSR spinning disk confocal microscope, and 32 *Z*-stacks with 0.34-μm steps were obtained with a 100× oil objective. IBA-1^+^ microglia and CD68^+^ lysosomes were then 3D reconstructed with Imaris software (Bitplane) using the surface rendering function, and synaptic puncta inside IBA-1^+^ phagocytes and CD68^+^ lysosomes were visualized and quantified using the spot rendering function. DAB-stained samples were imaged and quantitatively analyzed by the optical fractionator method (Stereo Investigator software, Microbrightfield).

### Behavioral tests.

Behavioral tests were conducted in MRL/lpr and pristane-induced lupus mice following previously established (9, 58) protocols, with some modifications. All behavioral tests were conducted between 9:00 and 18:00 hours. Behavior was monitored through a video camera positioned in front or on top of the testing apparatuses and was recorded and later analyzed with a video tracking system (TopScan software, CleverSys, Inc.) by a blinded, experienced researcher. For depression-like behavior characterization, a panel of behavioral tests, including the SPT, TST, and FST, was used (58). The detailed materials and methods of these tests are provided in the Supplemental Methods.

### Flow cytometry.

Briefly, single-cell suspensions of splenic cells from mice were prepared by mincing the spleens in PBS containing 0.5% FBS (Gibco Life Technologies) and 1% EDTA. PBMCs were isolated from EDTA-treated peripheral blood via Ficoll density gradient centrifugation. Erythrocytes were lysed using ACK lysis buffer, and the resulting cell suspension was washed and passed through 70-μm meshes. The cells were then counted and prepared for FACS analysis according to the flow cytometry protocols (28). For cytokine detection, 2 × 10^6^ single-cell suspensions were cultured in complete RPMI 1640 medium (Gibco) and stimulated with 50 ng/mL phorbol 12-myristate 13-acetate (Sigma-Aldrich), 1 μg/mL brefeldin A (Sigma-Aldrich), and 1 μg/mL ionomycin (Sigma-Aldrich) at 37°C with 5% CO_2_ for 5 hours. The cells were first stained for surface molecules (APC anti-mouse CD3e, 561826, BD Biosciences; FITC anti-mouse CD4, 561828, BD Biosciences; BV510 anti-mouse CD8α, 563068, BD Biosciences), fixed, permeabilized with a fixation/permeabilization kit (eBioscience), and then stained with an intracellular cytokine marker (IFN-γ–BV786). All of the cells were detected using a FACSCalibur flow cytometer (BD Biosciences) and analyzed via FlowJo software (Tree Star).

### Cytokine measurements.

The relative concentrations of inflammatory proteins in CSF were analyzed by a multiplex proximity extension assay with the provided Inflammation Probe Panel assay of 92 analytes (Olink Bioscience) using a Fluidigm Biomark reader (Fluidigm Corporation) following the vendor’s (LC-Bio Technology Co., Ltd.) recommended protocol. Among the 92 proteins, 54 were used in the analysis. The data are reported as normalized protein expression values (NPX, Olink Proteomics; arbitrary units on a log_2_ scale), as previously described (59). Significance was assessed using the *t* test (*P* < 0.05). Further details can be found in Supplemental Table 6.

### Statistics.

For all of the statistical analyses, GraphPad Prism 6.0 software was used. When possible, all analyses were performed by investigators who were blinded to the genotype and/or treatment group. The error bars represent the SEM in all of the figures. The analyses used in this study included 1-way ANOVA or Student’s 2-tailed *t* test. For ANOVAs, Tukey’s post hoc test or Šidák’s post hoc test was subsequently performed. Correlation analysis was performed via Spearman’s *r* test. A *P* value of less than 0.05 was considered significant.

### Study approval.

The present studies involving using human blood and CSF samples were reviewed and approved by The Ethics Committee for Human Investigation of the Nanjing Drum Tower Hospital (no. SC201700201), and written informed consent was obtained from all participants or their legally authorized representatives. All animal procedures were conducted in compliance with the NIH *Guide for the Care and Use of Laboratory Animals* (National Academies Press, 2011) and were approved by the Ethics Committee for Animal Research of Nanjing Drum Tower Hospital (no. 2019AE01084).

### Data availability.

All sequencing data are available in the NCBI’s Gene Expression Omnibus (GEO GSE154288 and GSE201282). All study data are included in the article, supplemental information, and the Supporting Data Values file. Other data that support the findings of this study are available from the corresponding author upon reasonable request.

## Author contributions

XH designed and performed most of the experiments, analyzed the data, and wrote the manuscript. DW and LC helped interpret the results. LC contributed to the isolation of the hUCMSCs. HS contributed to the flow cytometry. X Zheng, X Zhang, SZ, and JL contributed to the collection and assessment of the clinical samples. LC, JL, and TX provided clinical care, and referred patients. DW and ZH assisted in the experimental design and discussion. LS conceived the entire study and designed the experiments. All of the authors have read and approved the manuscript. The order of the co–first authors’ names was assigned on the basis of the academic contribution of each author. LS and ZH are co–senior and co–corresponding authors.

## Supplementary Material

Supplemental data

Unedited blot and gel images

Supporting data values

## Version 1 03/06/2025

In-Press Preview

## Version 2 04/22/2025

Electronic publication
