# Supplementary material for: MSC transplantation ameliorates depression in lupus by suppressing Th1 cell–shaped synaptic stripping
Source: JCI Insight. 2025 Mar 6;10(8):e181885. doi: 10.1172/jci.insight.181885 (PMC12016924; doi:10.1172/jci.insight.181885)
Supplement: Supplemental data [file jciinsight-10-181885-s144.pdf]

Supplementary Information for

MSC transplantation ameliorates depression in lupus by suppressing Th1 cell-shaped synaptic stripping

This file includes:

Supplementary Materials and Methods

Supplemental Figures, 1-11

Supplemental Tables, 1-6

Supplementary Materials and Methods

Study approval and human subjects

For individuals in the SLE group, the clinical diagnosis was assessed according to the American College of Rheumatology (ACR) revised SLE criteria (1) and the nomenclature and case definitions for NPSLE (2). Disease activity was measured with the SLEDAI (3). For healthy control subjects, the exclusion criteria included a history of neuropsychiatric disease, drug abuse, or head injury. Fluorochrome-labeled $CD4^{+}IFN\text{-}\gamma^{+}$ T cells and $CD8^{+}IFN\text{-}\gamma^{+}$ T cells among PBMCs were detected using a FACS Calibur flow cytometer (BD Biosciences), and the data were analyzed by FlowJo software (Tree Star). Human $CD4^{+}$ T cells were purified via magnetic cell sorting (Miltenyi Biotec), after which qPCR and immunoblotting were performed. Serum IFN-γ levels were measured by ELISA. For the in vitro coculture study, naive human $CD4^{+}$ T cells were purified via an EasySep kit (STEMCELL Technologies). CSF was collected from 14 patients with NPSLE and 9 SLE patients without NP manifestations (non-NP SLE patients). Six CSF samples from volunteers or epilepsy or encephalitis patients served as controls. Detailed information about the patients is provided in the Supplementary Information (Table S5).

Isolation, culture and transplantation of hUCMSCs

Ethical approval for this study was obtained from the Ethics Committee of the Affiliated Drum Tower Hospital of Nanjing University Medical School. Human umbilical cord (UC) samples were voluntarily donated by five individuals who delivered full-term infants via cesarean section. The UCs were collected and washed with ice-cold PBS. hUCMSCs were subsequently isolated and cultured in Dulbecco's modified Eagle's medium (DMEM)/F12 supplemented with 10% fetal bovine serum (Gibco Life Technologies) and 1% penicillin/streptomycin at 37 °C in 5% CO2 as described previously (4, 5). When they reached 80% confluence, the adherent cells

were harvested and subcultured for amplification. To delineate the stromal characteristics of the harvested cells, the immunophenotype (CD29, CD73, CD90, CD105, CD14, CD45, CD31, and HLA-DR expression) was assessed as previously described (5). For cryopreservation, the identified hUCMSCs from all donors were combined and resuspended in a cryoprotectant solution composed of 90% FBS and 10% dimethyl sulfoxide (DMSO). In this study, hUCMSCs between passages 3 and 6 (P3 to P6) were used for the experiments and distributed to the recipient mice in equal proportions. To study the effects of MSCT, 5-week-old MRL/mpj and MRL/lpr mice and 12-week-old (4 weeks following pristane injection) C57BL/6J and $Syn1^{Cre}$;$Ccl8^{fl/fl}$ mice were intravenously injected with hUCMSCs (5×10^5 in 500 μl PBS) or the same volume of PBS (without hUCMSCs) as a control (6). Behavioral tests were performed 3 weeks (MRL/lpr mice) or 6 weeks (pristane-induced lupus model) after MSCT. Brain tissues and sera were collected 24 hours after the behavioral tests.

Tissue collection and sample preparation

The mice were anesthetized with 3% isoflurane and then transcardially perfused with ice-cold PBS. One hemisphere was dissected to isolate the prefrontal cortex, hippocampus, cerebellum, and midbrain. A fraction of each sample was used for RNA extraction. For the RNA-seq analysis, a fraction of the hippocampal samples was used.

In vitro Th1 cell differentiation and culture with MSCs

Naive human $CD4^+$ T cells from PBMCs were purified using an EasySep kit (STEMCELL Technologies). Purified cells were cultured with plate-bound anti-CD3 (2.5 μg/ml; eBioscience) and anti-CD28 (5 μg/ml; eBioscience) for 5 days under Th1-polarizing conditions. For coculture analysis, sorted $CD4^+$ T cells were cultured with hUCMSCs in a Transwell system (0.4 μm pore size, Millipore) at a ratio of 10:1 (T cells:MSCs) under Th1-polarizing conditions.

Ex vivo isolation of microglia with microbeads

Ex vivo microglial isolation was performed as previously reported (7). The mice were deeply anesthetized and intracardially perfused with ice-cold PBS. The brains were extracted, minced and enzymatically digested in DMEM/F12 containing 2% FBS, 20 U/ml DNase I (Sigma-Aldrich) and 0.5 mg/ml collagenase type IV (Sigma-Aldrich) at 37 °C for 1 h with shaking. After careful homogenization with 19-G needles, the homogenates were pushed through a 70-μm strainer and then centrifuged at 500 × g for 10 min. The cell pellets were subsequently resuspended in 4 ml of a 37% Percoll solution (GE Healthcare). Next, the 70% Percoll (4 ml) solution containing the cells was transferred to a new 15 ml tube, and 4 ml of 37% Percoll was carefully overlaid on the layer containing cells, followed by a layer of 4 ml of 30% Percoll. The tube was centrifuged at 300 × g for 40 min at 18 °C without braking to remove myelin debris. Then, 2 ml of the 70%/37% interphase layer was collected, diluted with ice-cold PBS and subsequently centrifuged at 500 × g for 5 min at 4 °C. The pellets containing microglia were washed with PBS and then resuspended in MACS buffer. $CD11b^+$ microglia were isolated via manual MACS sorting (Miltenyi Biotec, 130-093-636) according to the manufacturer's instructions.

RNA sequencing

RNA sequencing of hippocampal tissue was performed as described in our previous study (8). Briefly, 3 μg of RNA from each sample was used as input material for the RNA sample preparations, and mRNA was purified from total RNA using poly-T oligo-conjugated magnetic beads. The sequencing libraries were generated via the NEBNext® UltraTM RNA Library Prep Kit for Illumina® (NEB, USA) according to the manufacturer’s recommendations (Novogene Co., Ltd.). The sequencing reads were aligned to the mouse reference genome mm10

(GRCm38.90) via STAR aligner (v2.5.1b) guided by the mouse GENCODE gene model release v15. HTSeq v0.6.0 was used to count the read numbers mapped to each gene. The FPKM value of each gene was subsequently calculated, the raw count data were normalized, and differential expression analysis was subsequently performed. Differentially expressed genes (DEGs) were defined as those with at least a 1.5-fold change in expression and adjusted $P < 0.05$ in comparisons of different genotypes.

RNA sequencing of isolated microglia was conducted as previously reported (9). Total RNA was extracted from microglia using TRIzol reagent (Thermo Fisher), and the mRNA was purified from the total RNA (5 mg) via Dynabeads Oligo (dT) (Thermo Fisher), fragmented into short fragments, and reverse transcribed to create cDNA templates using SuperScript™ II Reverse Transcriptase; these templates were subsequently used to synthesize the final cDNA libraries. Finally, 2×150-bp paired-end sequencing (PE150) was performed using the Illumina NovaSeq™ 6000 sequence platform (LC-Bio Technology Co., Ltd.) according to the vendor's recommended protocol. Reads obtained from the sequencing analyses were further filtered by Cutadapt and aligned to the murine reference genome using the HISAT2 package. DEGs were analyzed via DESeq2 software. Genes with a false discovery rate (FDR) < 0.05 and an absolute fold change ≥ 2 were considered DEGs. DEGs were then subjected to enrichment analyses of GO functions and KEGG pathways.

Brain immunohistochemical and immunofluorescence staining

As previously reported (8), the mice were deeply anesthetized and transcardially perfused with PBS, followed by perfusion with 4% paraformaldehyde (PFA) in PBS for fixation, postfixation in 4% PFA overnight, and cryoprotection in 20% sucrose. The brains were embedded in OTC and sectioned at a thickness of 25 μm with a freezing microtome. The sections

were preserved in a cryoprotectant (50% glycerol and 50% PBS) and stored at -20 °C. For H&E staining, the brains were embedded in paraffin and sectioned at 15 μm. For fluorescence immunostaining, free-floating sections were rinsed with PBS, permeabilized with PBS containing 0.3% Triton X-100 (PBST), blocked with blocking buffer (5% goat serum and 5% bovine serum albumin in PBST) at room temperature for 1 h, and incubated with primary antibodies overnight at 4 °C. After washing, the sections were incubated with secondary antibodies for 1 h at room temperature. The samples were then extensively washed and mounted in ProLong Diamond medium (Invitrogen, PK401). For immunochemical staining, endogenous peroxidases were neutralized (PBS/3% H2O2), and nonspecific binding was blocked. Then, the sections were stained with primary and secondary antibodies and visualized via 3'-diaminobenzidine immunostaining. The primary and secondary antibodies used in this study are listed in the Supplementary Information, Table S4. For neuron quantification, the sections were stained with Nissl staining solution (0.05% thionine/0.08 M acetate buffer, pH 4.5).

Golgi staining

For dendritic spine quantification, the sections were stained with an FD Rapid Golgi Stain Kit (FD NeuroTechnologies, PK401) according to the manufacturer's instructions. Golgi-stained neurons and dendritic segments from the cortex and hippocampus were imaged under a microscope (FV3000 Microscope, Olympus) with a 100 × objective. Dendritic branching and spines were analyzed via NIH ImageJ software.

Fluorescence in situ hybridization (FISH)

Fluorescent in situ hybridization (FISH) was performed on 8-μm-thick hippocampal sections from the formalin-fixed paraffin-embedded brain as previously described (10, 11). The sections were deparaffinized and then processed to detect the mRNA level and cell type specificity of

Ccl8 and *Eno2* in the mouse models of lupus according to the manufacturer's standard protocol (Gene Pharma). The sections were digested with proteinase K and heated to 78 °C for 8 min for denaturation. The RNA probes were incubated overnight at 37 °C with hybridization buffer containing 2 μM of a commercially available CY3-labeled CCL8 probe and 2 μM of a commercially available FAM-labeled *Eno2* probe. Finally, the sections were washed with PBS and mounted with ProLong Gold anti-fade reagent containing DAPI. Images of immunofluorescence staining were captured via a Zeiss LSM710 confocal microscope (Carl Zeiss Co.). The mouse *Ccl8* probe sequence, which was labeled with CY3 at the 5' end, was 5'-AGCCTTATCTGGCCCAGTCAGCTTCTC-3'. The 5' FAM-labeled mouse *Eno2* probe sequence was 5'-CACCGTCAGGTCATCGCCCACTATCT-3'. These probes were synthesized by GenePharma.

Quantitative RT-PCR

Real-time PCR was performed as described in our previous study using StepOnePlus Real-Time PCR Systems (Applied Biosystems) (8). Total RNA was extracted from tissues or cells using Trizol reagent (Vazyme Biotech, R401-01) according to the manufacturer's instructions. The RNA concentrations were adjusted to 1.0 μg/μl in nuclease-free water. For cDNA synthesis, reverse transcription was performed using a HiScript III RT SuperMix for qPCR Kit (Vazyme, R323-01). Next, the cDNAs were amplified and quantified using ChamQ SYBR qPCR Master Mix (Vazyme, Q341-02). *GAPDH* was used as an internal control. The sequences of the primers used for qPCR in this study (GenScript Biotech) are listed in Supplementary Table 3.

Enzyme-linked immunosorbent assay (ELISA)

The cell culture supernatant was collected and centrifuged at 1000 × g for 10 min at 4 °C. The supernatants were carefully collected and stored at -80 °C until assayed. Hippocampal tissues

(100 mg) were rinsed and homogenized in PBS (1 ml) before being stored overnight at -80 °C. After the cell membranes were disrupted by two freeze-thaw cycles, the homogenates were centrifuged at 5000 × g for 5 min at 4 °C, and the supernatants were collected (9). Human serum and CSF samples were collected, aliquoted, and stored at -80 °C until further analysis. The levels of anti-double-stranded DNA antibody (anti-dsDNA Ab) and cytokines (IFN-γ and/or CCL8) in the cell culture supernatant, serum, CSF or hippocampal extracts were measured using commercially available ELISA kits according to the manufacturers' instructions.

Mouse behavioral testing

Sucrose preference test (SPT) As previously described (9, 12, 13), the animals were first trained to consume a 1% w/v sucrose solution for 3 days from two different bottles. Twenty-four hours later, the animals were allowed free access to 1% w/v sucrose solution and tap water from two different bottles. The locations of the bottles (left or right) were switched across the study area to avoid a preference on the basis of the location of the bottles. Tap water and sucrose solution intake were measured after 24 hours by subtracting the final weight of the bottles from their initial weight. The preference for sucrose was then calculated as a percent preference = sucrose consumption/(sucrose + water consumption) × 100%. The tests were performed by an individual who was blinded to the animal's treatment status.

Forced swim test (FST) This procedure is used to induce a despair-like state and to test the effects of antidepressants on mice. The FST was performed as previously described, with slight modifications (12, 14). The mice were placed in a transparent glass cylinder (height, 35 cm; diameter, 17 cm) filled with water (23-25 °C) to a depth of 25 cm. The water depth was adjusted such that the animals had to swim or float without their hind limbs or tail touching the bottom. During testing (6-min trial), the duration of immobility (the time during which the subject made

only small movements necessary to keep their heads above water) within the final 4 min of the 6-min test was recorded using a video tracking system (TopScan software, CleverSys, Inc.). Each mouse was immediately removed from the cylinder and excluded from the study if it failed to swim or failed to keep its head above the water. After every trial, the water was changed, and the cylinder was rinsed with clean water.

Tail-suspension test (TST) This procedure is an alternative to the FST used to assess depression-like behavior. At the beginning of a trial, the mice were suspended by the tail (taped onto a suspension hook such that the animal would hang with its tail in a straight line) 50 cm above a flat surface. During testing (6-min trial), the duration of immobility (hanging passively without body movement) during the last 4 min of the 6-min test was monitored using a video tracking system and scored automatically (TopScan software, CleverSys, Inc.).

Open field The mouse open field chambers were made of Plexiglas and consisted of a square base (40 × 40 × 30 cm). For each test session, the mouse was allowed to explore the environment freely for 6 min. A computer-assisted video-tracking system (TopScan software, CleverSys, Inc.) was used to record the movement. The total distance traveled (cm) and the mean velocity (mm/s) during the test were used as measurements of general locomotor activity.

Assessment of lupus

Lupus was monitored by detecting albuminuria and autoantibody titers during the experiment, as described in our previous reports (8, 15). Urinary protein excretion was measured via a Bradford protein detection kit (Keygen Biotech, KGA801-804). Serum anti-dsDNA IgG titers were measured via ELISA (FUJIFILM, 631-02699) according to the manufacturer's instructions.

References

1. Hochberg, M.C. Updating the American College of Rheumatology revised criteria for the classification of systemic lupus erythematosus. *Arthritis & Rheumatism.* 1997;40(9):1725.

2. The American College of Rheumatology nomenclature and case definitions for neuropsychiatric lupus syndromes. *Arthritis & Rheumatism.* 1999;42(4):599-608.

3. Bombardier, C., et al. Derivation of the sledai. A disease activity index for lupus patients. *Arthritis & Rheumatism.* 1992;35(6):630-640.

4. Lei Q, et al. Extracellular vesicles deposit PCNA to rejuvenate aged bone marrow-derived mesenchymal stem cells and slow age-related degeneration. *Sci Transl Med*. 2021;13(578):eaaz8697.

5. Zhang, Z., et al. Human umbilical cord mesenchymal stem cells inhibit T follicular helper cell expansion through the activation of iNOS in lupus-prone B6.MRL-Faslpr mice. *Cell Transplant.* 2017;26(6):1031-1042.

6. Li, W., et al. Mesenchymal stem cells prevent overwhelming inflammation and reduce infection severity via recruiting $CXCR3^{+}$ regulatory T cells. *Clin Transl Immunology*. 2020;9(10):e1181.

7. Garber, C., et al. Astrocytes decrease adult neurogenesis during virus-induced memory dysfunction via IL-1. *Nat Immunol*. 2018;19(2):151-161.

8. Han X, et al. Neuronal NR4A1 deficiency drives complement-coordinated synaptic stripping by microglia in a mouse model of lupus. *Signal Transduct Target Ther*. 2022;7(1):50.

9. Zhang Y, et al. CircDYM ameliorates depressive-like behavior by targeting miR-9 to regulate microglial activation via HSP90 ubiquitination. *Mol Psychiatr*. 2020;25(6):1175-1190.

10. Lehrman, E.K., et al. CD47 protects synapses from excess microglia-mediated pruning during development. *Neuron*. 2018;100(1):120-134.

11. Di Liberto G, et al. Neurons under T cell attack coordinate phagocyte-mediated synaptic stripping. *Cell*. 2018;175(2):458-471.

12. Leng, L., et al. Menin deficiency leads to depressive-like behaviors in mice by modulating astrocyte-mediated neuroinflammation. *Neuron.* 2018;100(3):551-563.

13. Willner, P., et al. Reduction of sucrose preference by chronic unpredictable mild stress, and its restoration by a tricyclic antidepressant. *Psychopharmacology (Berl).* 1987;93(3):358-364.

14. Porsolt, R.D., Bertin, A., and Jalfre, M. Behavioral despair in mice: a primary screening test for antidepressants. *Arch Int Pharmacodyn Ther.* 1977;229(2):327-336.

15. Chen W, et al. Lipocalin-2 exacerbates lupus nephritis by promoting Th1 cell differentiation. *J Am Soc Nephrol*. 2020;31(10):2263-2277.

16. Ivashkiv LB. IFNgamma: signalling, epigenetics and roles in immunity, metabolism, disease and cancer immunotherapy. *Nat Rev Immunol*. 2018;18(9):545-558.

Supplemental Figure 1

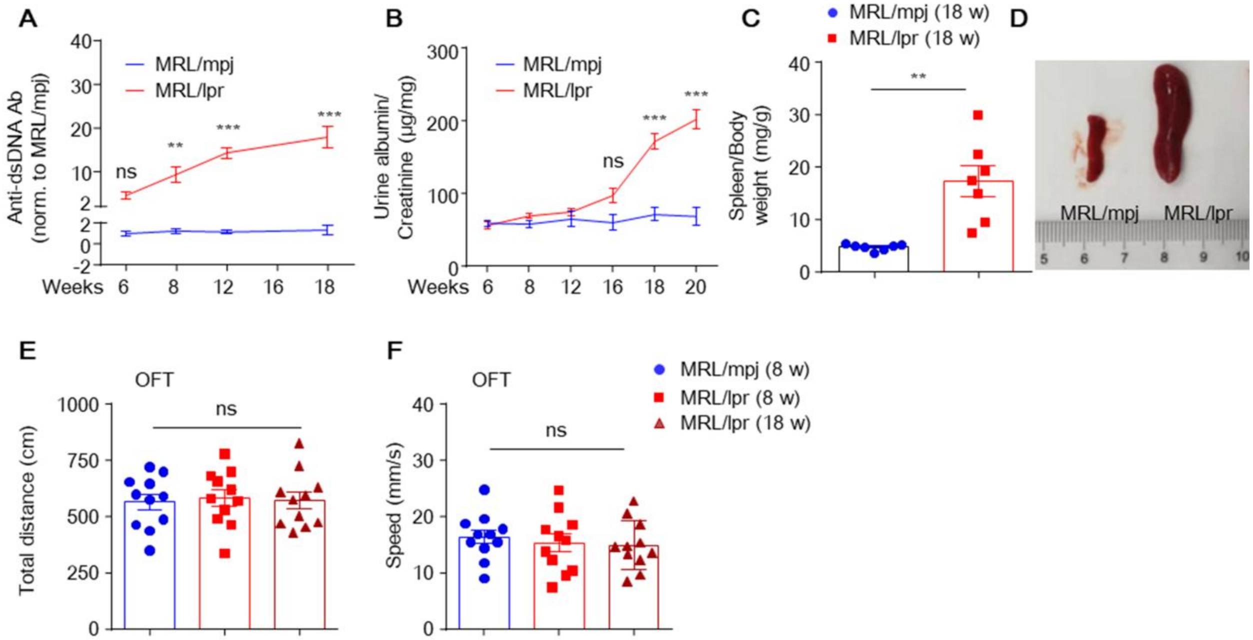

Supplemental Figure 1. Characteristics of typical SLE lesions and the general motion of MRL/lpr mice, related to Figure 1.

(**A**) Serum anti-dsDNA IgG titers in age-matched MRL/mpj and MRL/lpr mice were examined via ELISA (*n* = 4-5 mice/group). (**B**) Albuminuria levels in age-matched MRL/mpj and MRL/lpr mice (*n* = 9-10 mice/group). (**C** and **D**) Spleen/body weight ratios and sizes of the spleens from the indicated mice (*n* = 7 mice/group). (**E** and **F**) Evaluation of the general locomotion of MRL/lpr and MRL/mpj mice (*n* = 11 mice/group) at 8 and 18 weeks of age via the OFT. The data are presented as the mean ± SEM. ** $P < 0.01$; *** $P < 0.001$; ns, not significant; unpaired Student's *t* test or one-way ANOVA followed by Tukey's or Sidak's post hoc test. OFT, open field test.

Supplemental Figure 2

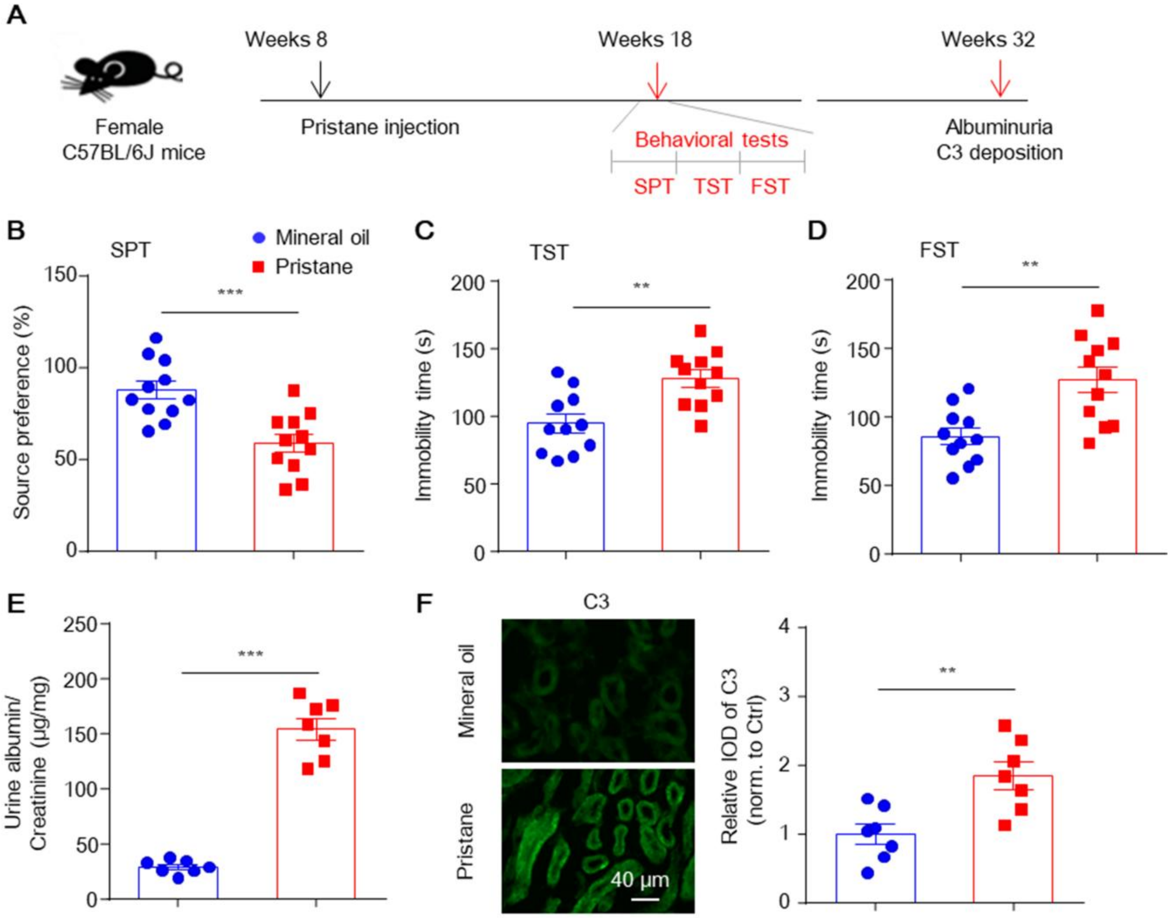

Supplemental Figure 2. Pristane-induced lupus mice exhibit increased depression-like behavior before the appearance of overt lupus nephritis, related to Figure 1.

(**A**) Timeline of the experimental procedure for the pristane-induced lupus model. (**B**-**D**) Evaluation of depression-like behavior in pristane- and mineral oil-injected mice (n = 11 mice/group). Ten weeks after pristane or mineral oil injection, the mice were subjected to the SPT (**B**), TST (**C**), and FST (**D**). Twenty-four weeks after pristane or mineral oil injection, albuminuria (**E**) and C3 deposition in the kidney (**F**) were evaluated (n = 7). The data are presented as the mean ± SEM. ** $P < 0.01$; *** $P < 0.001$ according to unpaired Student's t test. SPT, sucrose preference test; TST, tail suspension test; FST, forced swim test; IOD, integrated optical density; Ctrl, control.

Supplemental Figure 3

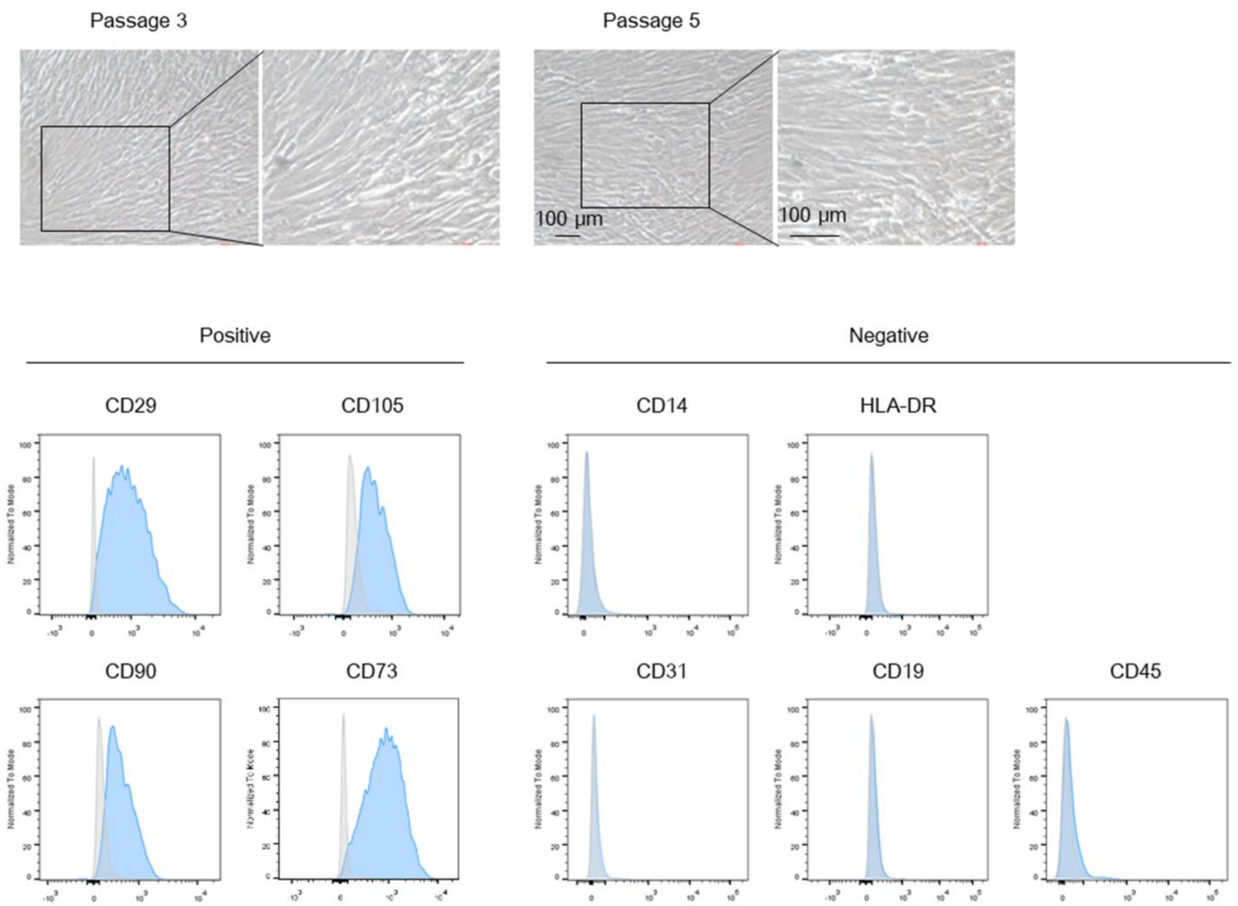

Supplemental Figure 3. Characteristics, including the morphology and phenotype, of hUCMSCs, related to Figure 1.

(**A**) Representative optical images showing the morphology of P3 and P5 primary hUCMSCs derived from the primary human umbilical cord. Scale bar, 100 µm. (**B**) Flow cytometry analysis showing that hUCMSCs were positive for mesenchymal lineage markers (CD29, CD105, CD90 and CD73), negative for hematopoietic and endothelial markers (CD14, CD31, CD19, and CD45), and negative for HLA-DR.

Supplemental Figure 4

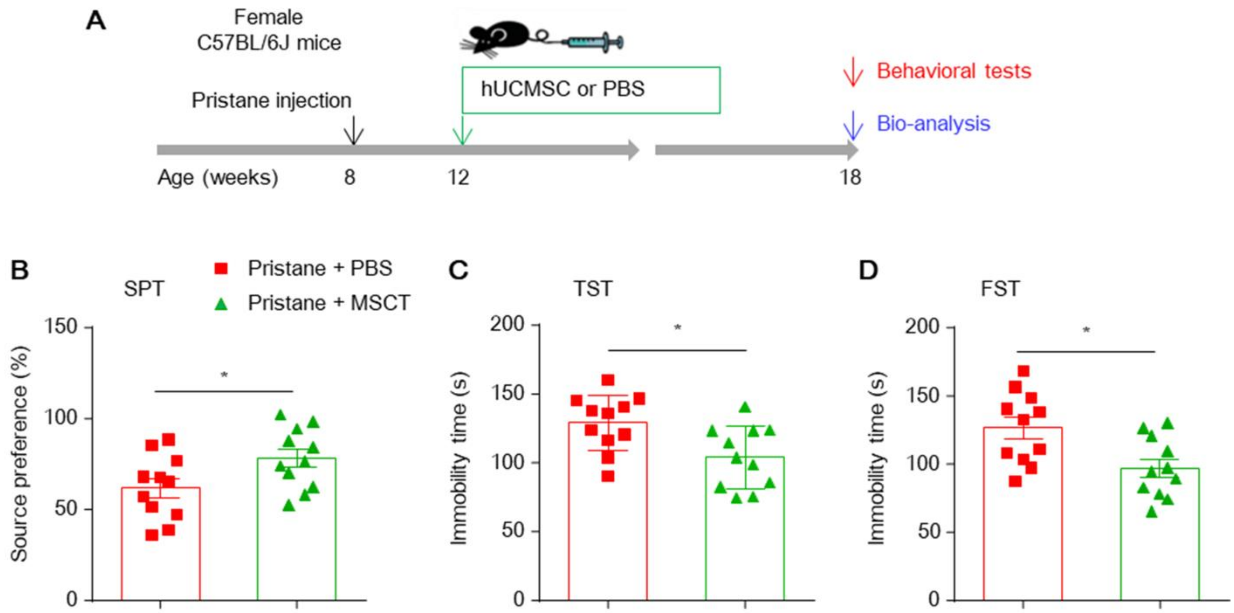

Supplemental Figure 4. MSCT alleviates depression in recipient pristane-induced lupus mice, related to Figure 1.

(**A**) Experimental protocol for the treatment of pristane-induced lupus mice (*n* = 11 mice/group) by MSCT. (**B-D**) Effects of MSCT on depression-like behavior in pristane-induced lupus model mice. Eight-week-old C57BL/6J mice were intraperitoneally (i.p.) injected with pristane (500 μl). Four weeks later, the mice were intravenously injected with hUCMSCs (5×10^5 cells in 500 μl of PBS) or PBS (as a control). The SPT (**B**), TST (**C**), and FST (**D**) were performed 6 weeks after MSCT. The data are presented as the mean ± SEM. * $P < 0.05$ according to unpaired Student's *t* test. SPT, sucrose preference test; TST, tail suspension test; FST, forced swim test.

266 **Supplemental Figure 5**

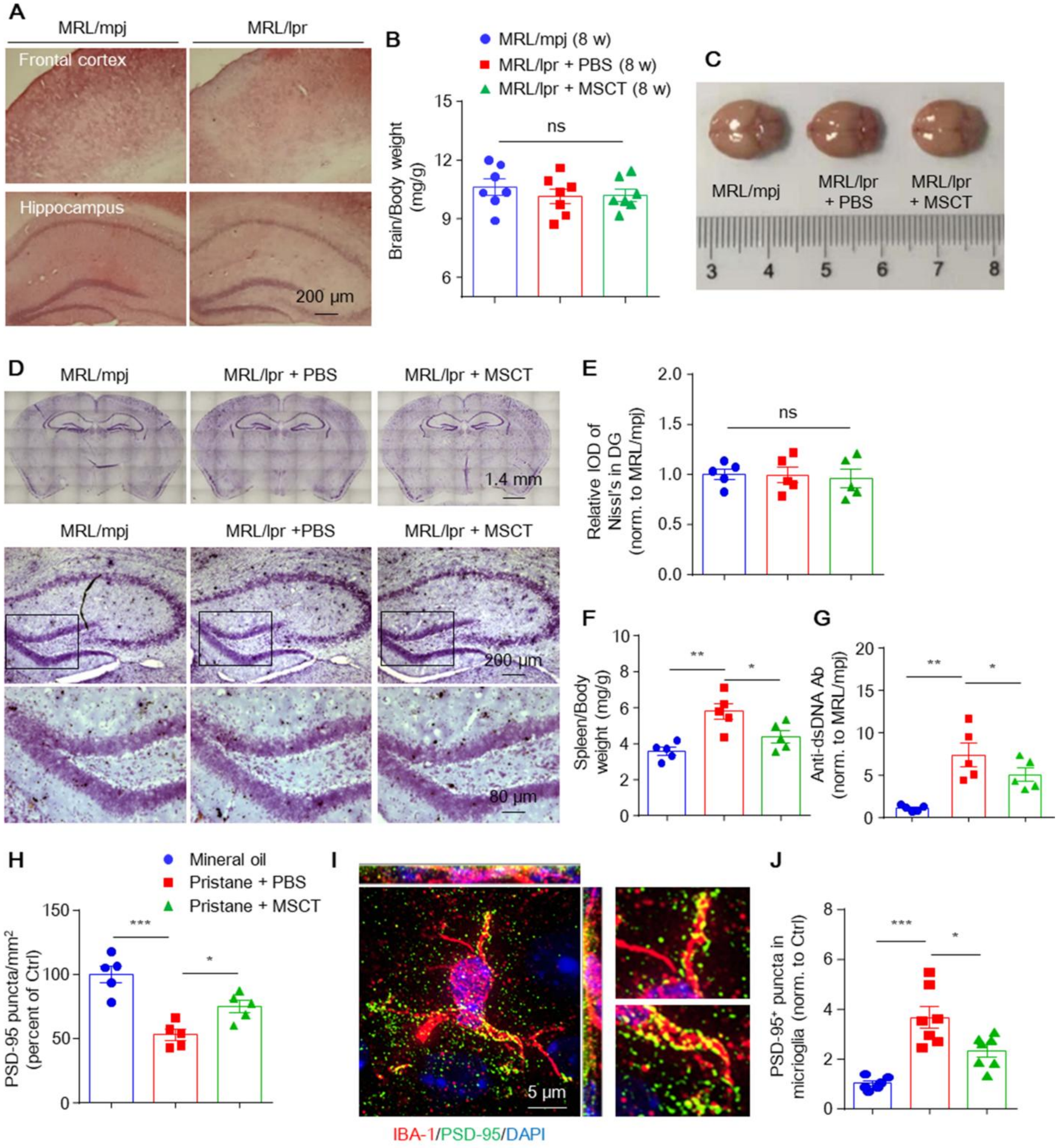

267 **Supplemental Figure 5. No marked anatomical abnormalities or neuron loss are observed**
268 **in young MRL/lpr mice, and dendritic loss is observed in the brains of mice with pristane-**
269 **induced lupus, related to Figure 2.**

(**A**) Representative images of H&E stained samples showing no evidence of gross anatomical abnormalities in MRL/lpr mice at 8 weeks. Scale bar, 200 μm. (**B** and **C**) Brain/body weight ratios and brain sizes of MRL/mpj and MSCT/PBS treated MRL/lpr mice (*n* = 7 mice/group). (**D** and **E**) Immunostaining and quantification of $Nissl^+$ neurons within the hippocampus (with higher magnification images of the dentate gyrus shown) in 8-week-old MRL/mpj and MSCT/PBS treated MRL/lpr mice (*n* = 5 mice/group). The scale bars are indicated. (**F** and **G**) Spleen/body weight ratios and serum anti-dsDNA antibody levels in 8-week-old MRL/mpj and MSCT/PBS treated MRL/lpr mice (*n* = 5 mice/group). (**H**) Quantification of postsynaptic density (PSD-95) in hippocampal sections from each treatment group (*n* = 5 mice/group, with an average of 3-4 slices/mouse). (**I** and **J**) Immunostaining and quantification of postsynaptic (PSD-95, green) puncta in hippocampal microglia (IBA-1, red) from each treatment group (*n* = 6-7 mice/group, with an average of 3-5 cells/mouse). Scale bar, 5 μm. The data are presented as the mean ± SEM. * $P < 0.05$; ** $P < 0.01$; *** $P < 0.001$; ns, nonsignificant; one-way ANOVA followed by Tukey's or Sidak's post hoc test. IOD, integrated optical density; Ctrl, control.

284 **Supplemental Figure 6**

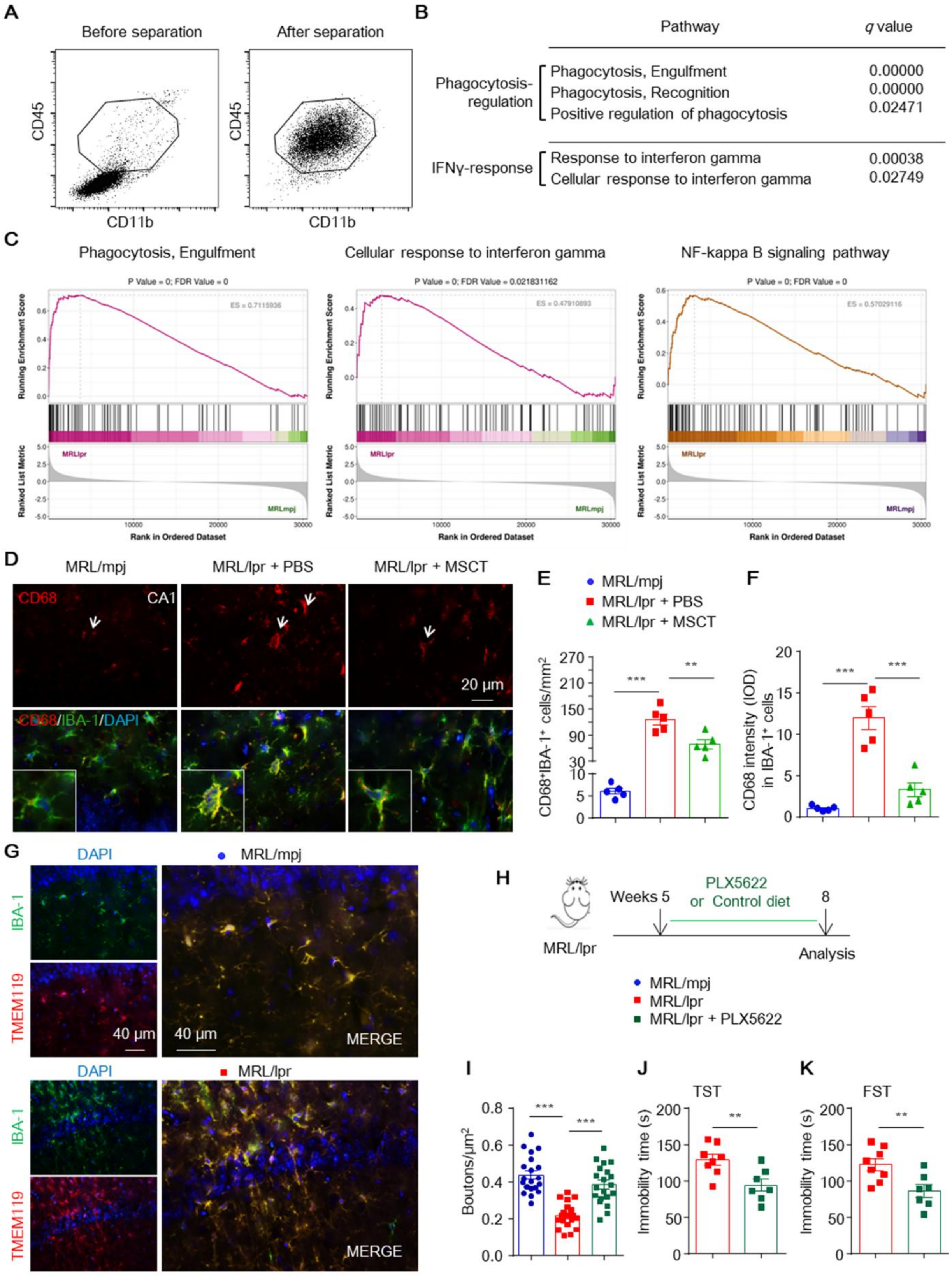

Supplemental Figure 6. MSCT rescues genetic and functional changes in microglia from MRL/lpr mice, related to Figure 2.

(**A**) Representative dot plots of CD45 and CD11b labeling of microglia (gated in black frame) collected after microbead kit separation. (**B**) GO-seq analysis of the phagocytosis-regulating and IFN-γ-responsive gene sets in microglia isolated from MRL/lpr vs MRL/mpj mice. (**C**) GSEA revealed that phagocytosis, response to IFN-γ, and NF-κB signaling pathway gene sets were significantly enriched in MRL/lpr microglia compared with MRL/mpj microglia (|NES|>1, NOM *P* value < 0.05, FDR q value < 0.25). (**D-F**) Representative images and quantification of CD68$^+$IBA-1$^+$ phagocytes (**E**) and CD68 staining intensity (**F**) in hippocampal sections from the indicated mice (*n* = 5 mice/group). Scale bar, 20 μm. (**G**) Representative brain sections coimmunostained for the microglia-specific marker TMEM119 and IBA-1 showing an abundance of amoeboid phagocytes (IBA-1) enriched in TMEM119 expression in the evaluated lupus mice. (**H**) Pharmacological depletion of microglia in MRL/lpr mice. MRL/lpr mice received a control or PLX5622 diet from 5 to 8 weeks of age. (**I**) Quantification of SYP$^+$ boutons in the hippocampi of the indicated groups. (**J** and **K**) Depression-like behaviors were assessed. The TST (**J**) and FST (**K**) were performed after 3 weeks of PLX5622 treatment. (*n* = 7-8 mice/group). The data are presented as the mean ± SEM. ** *P* < 0.01; *** *P* < 0.001; one-way ANOVA followed by Tukey's post hoc test or unpaired Student's *t* test. TST, tail suspension test; FST, forced swim test; IOD, integrated optical density.

Supplemental Figure 7

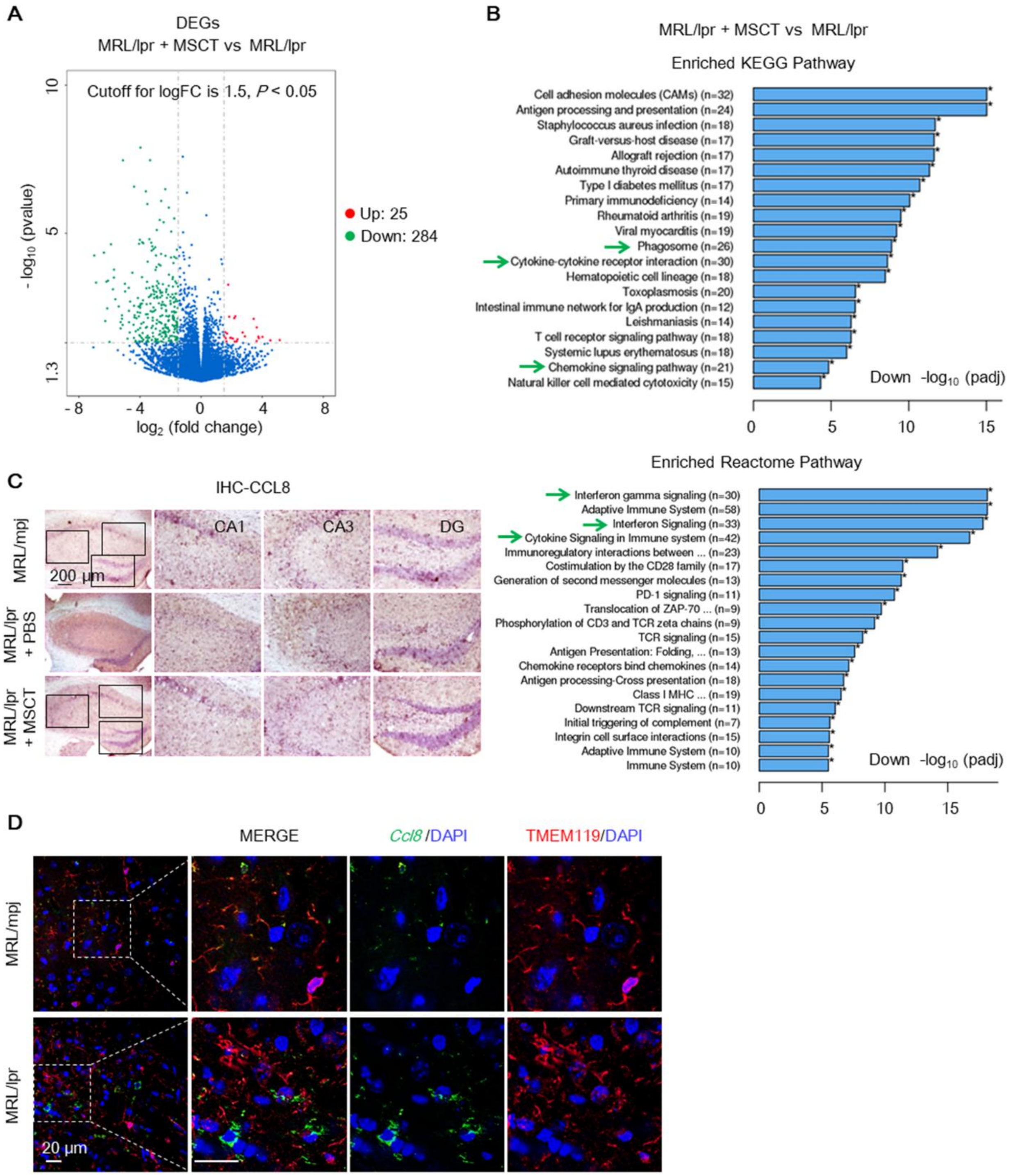

Supplemental Figure 7. RNA-seq and Gene Ontology analyses of the hippocampus of MRL/lpr mice, related to Figure 3.

(**A**) Volcano plot of genes with significant fold changes induced by MSCT in MRL/lpr mice quantified via RNA-seq. Only genes identified with a fold change ≥ 1.5 and a P value < 0.05 were regarded as significantly altered. (**B**) Enriched KEGG and Reactome pathway analyses revealed that MSCT reversed the changes in pathways in the hippocampi of MRL/lpr mice identified by sequencing. (**C**) Representative image of CCL8 immunostaining in hippocampal sections from 8-week-old MRL/mpj and MSCT- or PBS-treated MRL/lpr mice. Scale bar, 200 µm. (**D**) RNAscope in situ hybridization revealed that elevated *Ccl8* expression is rarely detected in microglia (colabeled with anti-TMEM119 antibody) in hippocampal sections from 8-week-old MRL/lpr mice. Scale bars, 20 µm. vs, versus.

316 **Supplemental Figure 8**

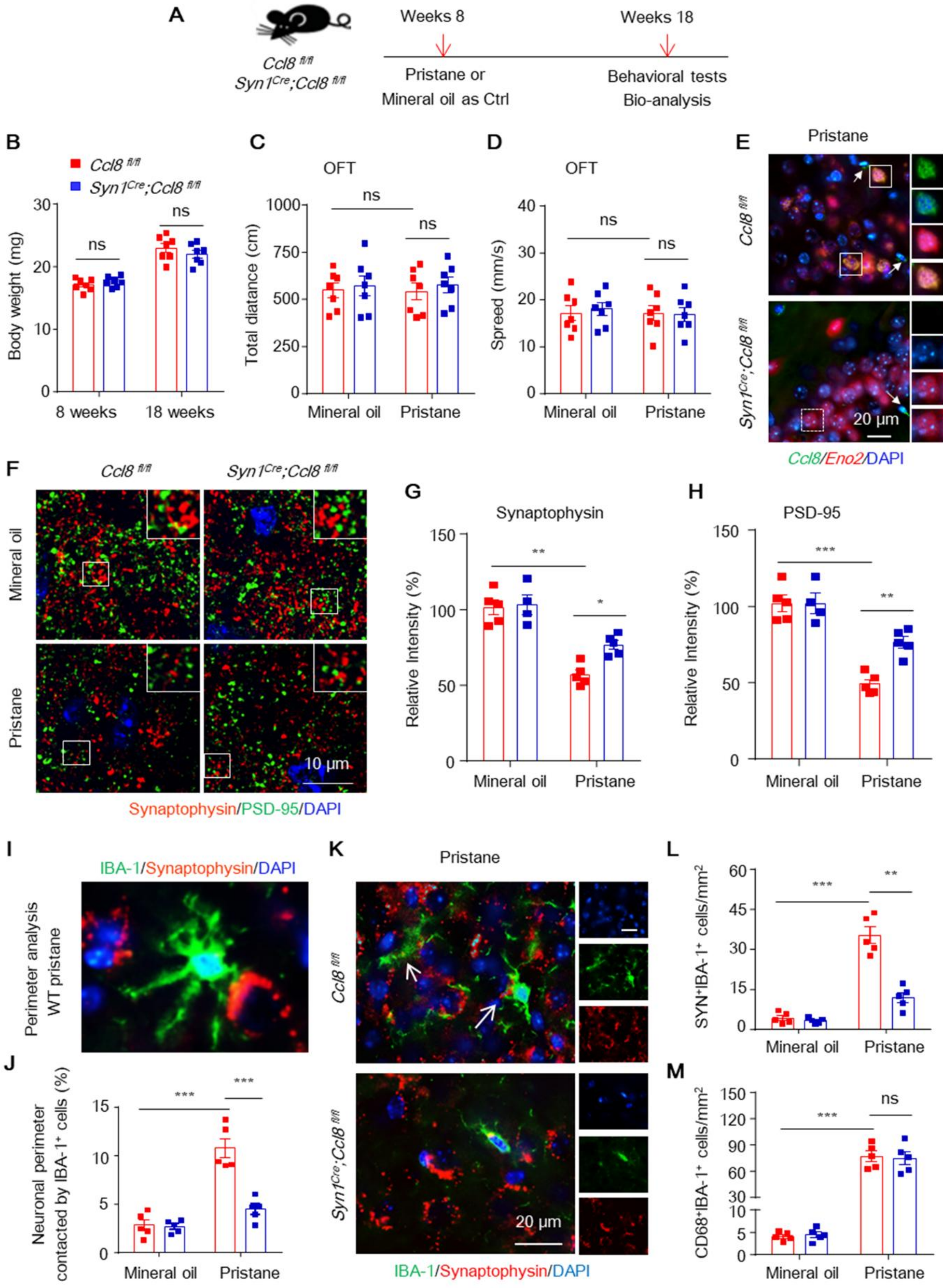

Supplemental Figure 8. Neuronal *Ccl8* deficiency prevents depression in pristane-induced lupus mice, related to Figure 3.

(**A**) Experimental outline describing model induction and analysis. (**B**) Body weights of mineral oil (used as a control, Ctrl) or pristane treated $Ccl8^{fl/fl}$ and $Syn1^{Cre}$;$Ccl8^{fl/fl}$ mice. (**C** and **D**) General locomotor activity of each treatment group (n = 7 mice/group). Ten weeks after pristane injection, the mice were subjected to the OFT. (**E**) RNAscope in situ hybridization confirmed abrogated expression of *Ccl8* mRNAs in the neurons (colabeled with the *Eno2* probe) of pristane treated $Syn1^{Cre}$;$Ccl8^{fl/fl}$ mice. Scale bar, 20 µm. The square indicates $Ccl8^{+}Eno2^{+}$ neurons, the dashed square indicates $Ccl8^{-}Eno2^{+}$ neurons, whereas the arrowhead indicates $Ccl8^{+}Eno2^{-}$ nonneuronal cells. (**F**-**H**) Immunostaining and quantification of presynaptic (synaptophysin, red) and postsynaptic (PSD-95, green) boutons in hippocampal sections from each treatment group (n = 4-5 mice/group, with an average of 3-4 slices/mouse). Scale bar, 10 µm. (**I** and **J**) Images and proportions of neuronal perimeter contacted by IBA-1^{+} cells in hippocampal slices (n = 5 mice/group). (**K**) Images of synaptic structures (SYP^{+}) localized in contact with CNS phagocytes (IBA-1) in brain sections from the indicated mice. (**L**) The number of SYP^{+}IBA-1^{+} phagocytes per mm^2 is shown. The data are presented as the mean ± SEM. (**M**) Quantification of $CD68^{+}$IBA-1^{+} phagocytes in hippocampal sections from the indicated mice (n = 5 mice/group). * $P < 0.05$; ** $P < 0.01$; *** $P < 0.001$; ns, not significant; one-way ANOVA followed by Tukey's or Sidak's post hoc test.

336 **Supplemental Figure 9**

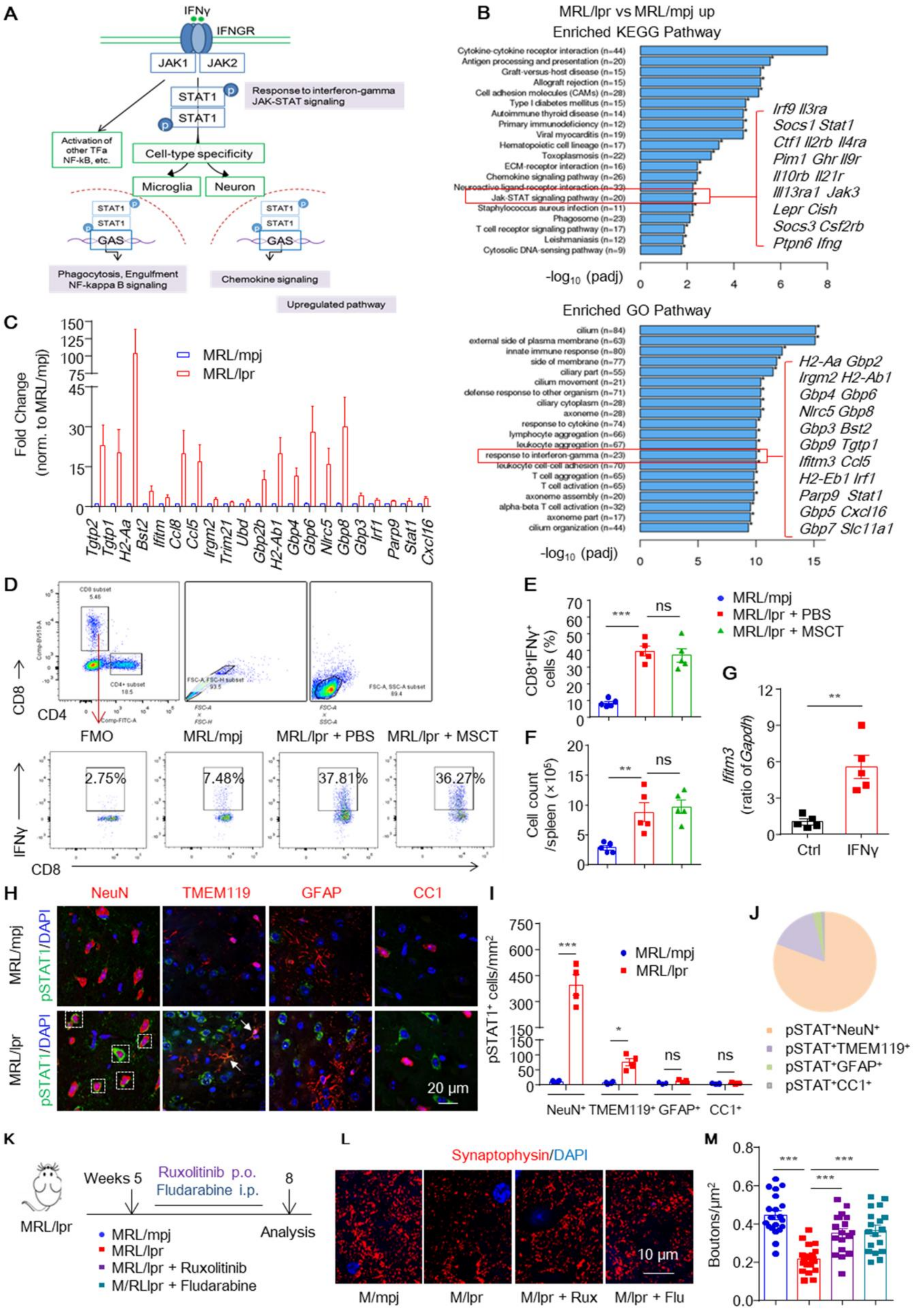

Supplemental Figure 9. IFN-γ/JAK-STAT1 signaling contributes to MSCT-mediated neuroprotection in recipient MRL/lpr mice, related to Figure 4.

(**A**) Schematic of the IFN-γ signaling pathway (16) and pathway map of genes activated in response to IFN-γ that are differentially expressed in lupus brain. (**B**) KEGG and GO analyses of the identified upregulated gene sets in hippocampal tissues from MRL/lpr mice. (**C**) RNA-seq analysis revealed elevated expression of interferon-responsive genes in microglia isolated from MRL/lpr mice compared with those isolated from MRL/mpj mice. (**D**-**F**) Gating schemes for flow cytometry analysis of CD3$^+$CD4$^+$IFN-γ^+ and CD3$^+$CD8$^+$IFN-γ^+ cells in the mouse spleen. The percentages (**E**) and numbers (**F**) of splenic CD8$^+$IFN-γ^+ cells in the indicated groups (n = 5 mice/group). (**G**) Hippocampal neurons were treated with IFN-γ (10 ng/ml) and *Ifitm3* expression was assessed 6 hours after treatment. (**H** and **I**) Representative images of brain sections coimmunostained for pSTAT1 together with neuronal (NeuN$^+$) and nonneuronal markers (TMEM119 for microglia, GFAP for astrocytes, and CC1 for oligodendrocytes) and quantification of positive cells in the hippocampi of the indicated groups (n = 3-4 mice per group). Scale bar, 20 μm. The dashed square indicates pSTAT1$^+$ neurons whereas the arrowhead indicates pSTAT1$^+$ microglia. (**J**) Quantification analysis of the fraction of pSTAT1$^+$ cells that coexpressed the indicated markers in the hippocampi of 8-week-old MRL/lpr mice. (**K**-**M**) Ruxolitinib (Rux, p.o.) or fludarabine (Flu, i.p.) was given to MRL/lpr mice from 5 to 8 weeks of age. SYP immunoreactivity were analyzed in 18-20 CA1 neurons from 3-4 mice per group (**L** and **M**). Scale bar, 10 μm. The data are presented as the mean ± SEM. * $P < 0.05$; ** $P < 0.01$; *** $P < 0.001$; ns, not significant; unpaired Student's t test or one-way ANOVA followed by Tukey's or Sidak's post hoc test.

Supplemental Figure 10

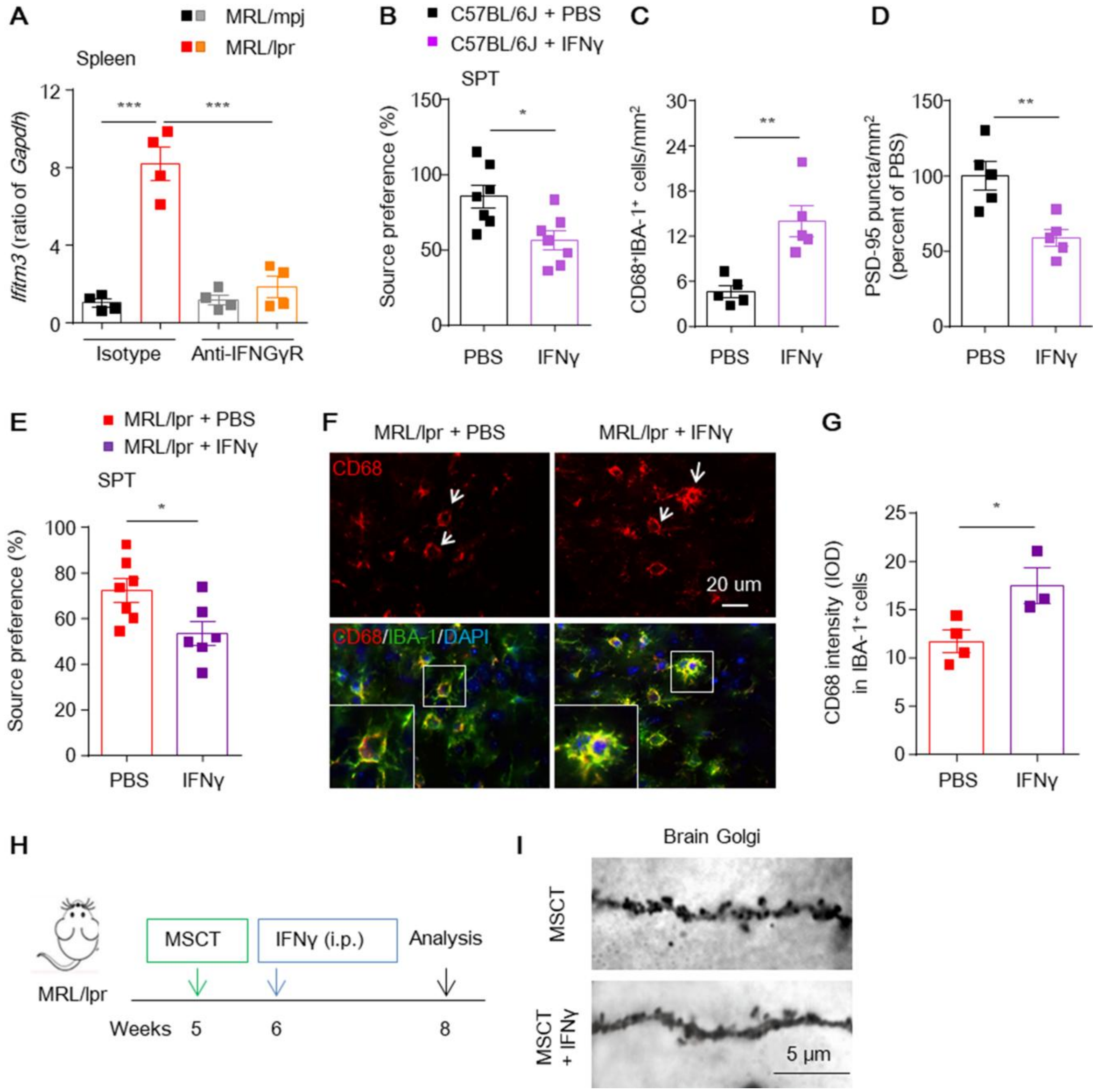

Supplemental Figure 10. Blockade of IFN-γ signaling rescues depression phenotypes in MRL/lpr mice, related to Figure 5.

(**A**) qPCR analysis of *Ifitm3* expression in the spleens of the indicated mice (*n* = 4 mice/group). (**B**) Depression-like behaviors (SPTs) were assessed in PBS or IFN-γ treated C57BL/6J mice (*n* = 7 mice/group). (**C** and **D**) Quantification of CD68^{+}IBA-1^{+} phagocytes (**C**) and PSD-95 staining intensity (**D**) in the hippocampi of the indicated mice (*n* = 5 mice/group). (**E**) Depression-like

behaviors (SPTs) were assessed in PBS or IFN-γ treated MRL/lpr mice (8 weeks old, *n* = 6-7 mice/group). (**F** and **G)** Representative images and quantification of CD68 staining intensity in hippocampal sections from the indicated mice (*n* = 3-4 mice/group). Scale bar, 20 μm. (**H**) Experimental outline describing model induction and analysis. (**I**) Image of Golgi-stained dendritic spines from DG granule neurons in MSCT (alone or in combination with IFN-γ) treated MRL/lpr mice. Scale bar, 5 μm. The data are presented as the mean ± SEM. * $P < 0.05$; ** $P < 0.01$; *** $P < 0.001$; unpaired Student's *t* test or one-way ANOVA followed by Tukey's post hoc test. SPT, sucrose preference test; IOD, integrated optical density.

Supplemental Figure 11

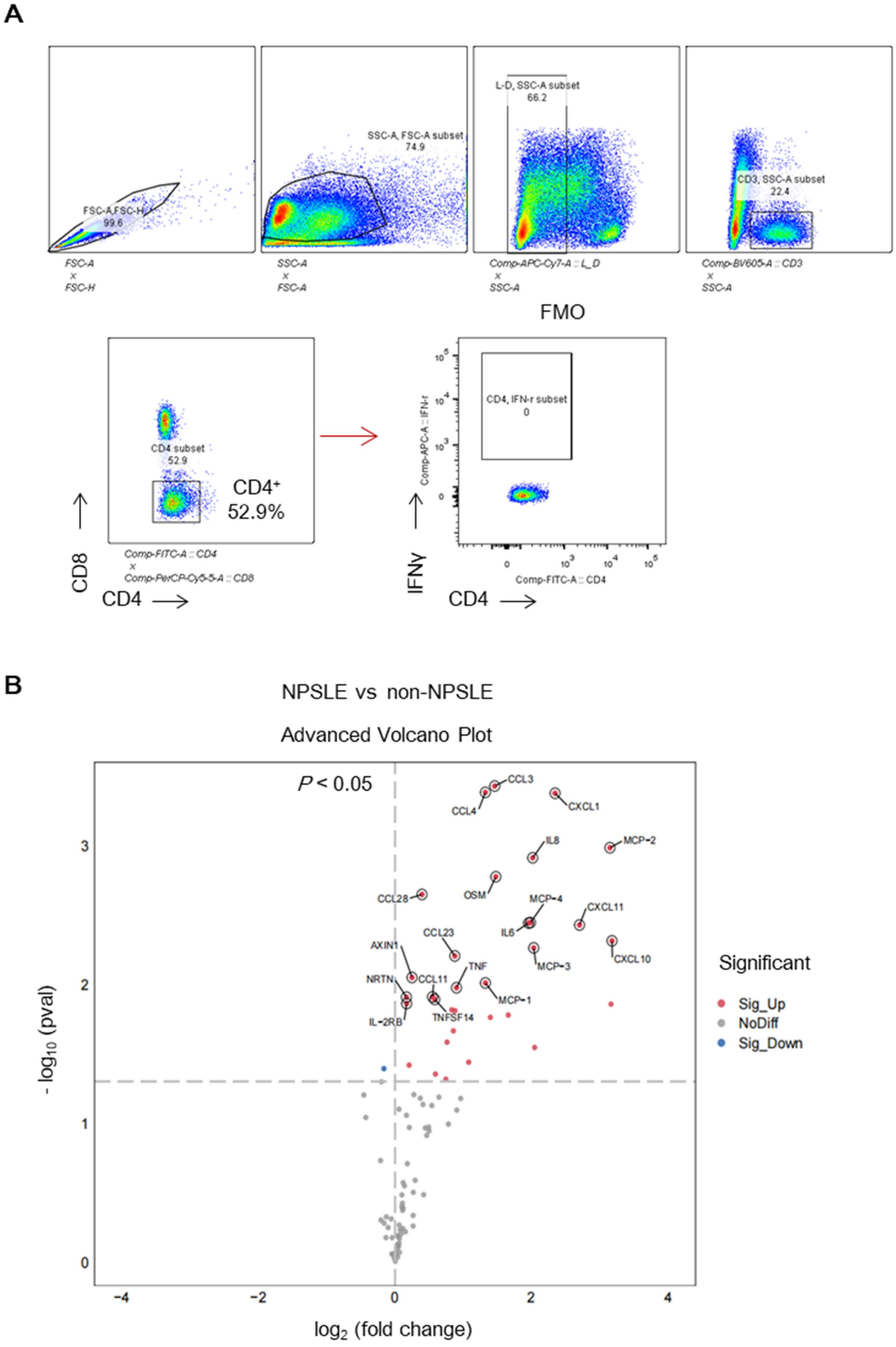

Supplemental Figure 11. Flow cytometry analysis of CD4$^+$IFN-γ^+ T cells in human PBMCs (A), and volcano plot of O-link-quantified differentially expressed proteins between patients with NPSLE and those with non-NPSLE (B), related to Figure 6.

Supplemental Table 1. Enrichment analysis of genes altered in MRL/lpr compared with MRL/mpj mice by GSEA with KEGG modules. The top 20 pathway lists were generated with the genes with greater expression in MRL/lpr than in MRL/mpj (≥1.5-fold changes and *P* value < 0.05).

MRL/mpj < MRL/lpr (KEGG)

Pathways	genes	P value	Select genes within pathway
Cytokine-cytokine receptor interaction	44	5.74E-11	Ccl8/Kdr/Cxcl13/Cxcl10/Ccl5/Ngfr/Ccr1/Tnfsf10/Ltb/Ccr2/Il3ra/Tgfbr2/Cxcl11/Kitl/Il18r1/Tnfrsf1b/Ctf1/Cxcr4/Ccl3/Cxcl16/Il2rb/Il4ra/Ghr/Pdgfc/Il9r/Il10rb/Bmp2/Met/Il21r/Il13ra1/Cxcr6/Lepr/Ccr5/Csf2rb/Cxcl9/Edar/Ccr7/Xcr1/Ccl28/Tnfrsf14/Cxcr3/Cd27/Tnfsf8/Ifng
Antigen processing and presentation	20	3.22E-08	H2-Aa/H2-Ab1/B2m/Cd74/H2-Eb1/H2-T22/H2-T9/Tap1/H2-T23/Cd8a/Cd4/Ciita/Tap2/H2-M2/Tapbp/H2-DMb2/H2-T24/H2-DMb1/H2-K1/Ifng
Allograft rejection	15	1.64E-07	H2-Aa/H2-Ab1/H2-Eb1/H2-T22/H2-T9/H2-T23/H2-M2/H2-DMb2/H2-T24/Cd28/Prf1/Gzmb/H2-DMb1/H2-K1/Ifng
Graft-versus-host disease	15	1.64E-07	H2-Aa/H2-Ab1/H2-Eb1/H2-T22/H2-T9/H2-T23/H2-M2/H2-DMb2/H2-T24/Cd28/Prf1/Gzmb/H2-DMb1/H2-K1/Ifng
Cell adhesion molecules (CAMs)	28	2.47E-07	H2-Aa/H2-Ab1/Cldn5/Ptprc/H2-Eb1/H2-T22/H2-T9/Itgal/H2-T23/Sdc2/Cldn3/Cd8a/Cd2/Cd274/Cd4/H2-M2/Spn/H2-DMb2/Itgb7/Cldn19/H2-T24/Cd28/Pdcd1/F11r/H2-DMb1/H2-K1/Cdh4/Cd6
Type I diabetes mellitus	15	1.08E-06	H2-Aa/H2-Ab1/H2-Eb1/H2-T22/H2-T9/H2-T23/H2-M2/H2-DMb2/H2-T24/Cd28/Prf1/Gzmb/H2-DMb1/H2-K1/Ifng
Viral myocarditis	19	1.74E-06	H2-Aa/H2-Ab1/Casp9/Myh7/H2-Eb1/H2-T22/H2-T9/Itgal/H2-T23/Rac2/H2-M2/Casp3/H2-DMb2/H2-T24/Cd28/Prf1/H2-DMb1/H2-K1/Casp8
Primary immunodeficiency	12	1.92E-06	Ptprc/Tap1/Cd3e/Cd8a/Cd3d/Cd4/Ciita/Tap2/Cd79a/Lck/Cd19/Jak3
Autoimmune thyroid disease	14	2.05E-06	H2-Aa/H2-Ab1/H2-Eb1/H2-T22/H2-T9/H2-T23/H2-M2/H2-DMb2/H2-T24/Cd28/Prf1/Gzmb/H2-DMb1/H2-K1
Primary immunodeficiency	12	3.59E-05	Ptprc/Tap1/Cd3e/Cd8a/Cd3d/Cd4/Ciita/Tap2/Cd79a/Lck/Cd19/Jak3
Autoimmune thyroid disease	14	5.42E-05	H2-Aa/H2-Ab1/H2-Eb1/H2-T22/H2-T9/H2-T23/H2-M2/H2-DMb2/H2-T24/Cd28/Prf1/Gzmb/H2-DMb1/H2-K1
Hematopoietic cell lineage	17	2.50E-05	Cd3g/H2-Eb1/Cd24a/Cd3e/Il3ra/Cd8a/Cd2/Cd3d/Cd5/Kitl/Cd4/ Cd44/Itga2/ Cd59b/Il4ra/Il9r/Cd19
Toxoplasmosis	22	5.87E-05	H2-Aa/H2-Ab1/Casp9/Lama3/Igtp/Pla2g5/H2-Eb1/Bcl2/Tlr2/Ciita/Socs1/Stat1/Casp3/H2-DMb2/Il10rb/Pla2g2d/Lamc2/Ccr5/H2-DMb1/Lamb3/Casp8/Ifng
Chemokine signaling pathway	26	2.65E-04	Ccl8/Cxcl13/Cxcl10/Ccl5/Grk5/Ccr1/Adcy7/Rac2/Ccr2/Cxcl11/Stat1/Plcb4/Cxcr4/Hck/Ccl3/Cxcl16/Jak3/Cxcr6/Ccr5/Gnb4/Cxcl9/Ccr7/Xcr1/Prkcd/Ccl28/Cxcr3
ECM-receptor interaction	16	2.71E-04	Spp1/Lama3/Col6a3/Thbs4/Sdc2/Sv2c/Thbs1/Col5a2/Cd44/Itga2/Itgb7/Col4a4/Col5a3/Lamc2/Lamb3/Itgb6
Jak-STAT signaling pathway	20	4.76E-04	Irf9/Il3ra/Socs1/Stat1/Ctf1/Il2rb/Il4ra/Pim1/Ghr/Il9r/Il10rb/Il21r/Il13ra1/Jak3/Lepr/Cish/Socs3/Csf2rb/Ptpn6/Ifng
Neuroactive ligand-receptor interaction	33	4.82E-04	Htr2c/Avpr1a/Crhr2/Adra2b/Trhr/Grm8/Npffr1/Drd2/Grik3/Glp1r/S1pr3/Mc3r/Tspo/Adra2a/Gabre/Galr1/Glra1/Calcrl/Lpar6/Ghr/Gpr35/Htr7/Lhb/Sstr5/Ptafr/Oxtr/Grm4/Gpr50/Lepr/Agtr1a/Oprk1/Grin2d/Hrh1
Staphylococcus aureus infection	11	5.21E-04	H2-Aa/H2-Ab1/H2-Eb1/Itgal/C1s1/H2-DMb2/C1ra/Ptafr/Fcgr4/H2-DMb1/Hc
Phagosome	23	7.37E-04	H2-Aa/H2-Ab1/H2-Eb1/H2-T22/H2-T9/Thbs4/Tap1/H2-T23/Cybb/Clec7a/Thbs1/Cyba/Tlr2/Tap2/H2-M2/Itga2/H2-DMb2/H2-T24/C1ra/Fcgr4/H2-DMb1/Ncf2/H2-K1
T-cell receptor signaling pathway	17	0.001353	Cd3g/Ptprc/Tec/Cd3e/Cd8a/Cd3d/Cd4/Lat/Cd247/Cd28/Pdcd1/Lck/Card11/Prkcq/Ptpn6/Ifng/Grap2
Leishmaniasis	12	0.001616	H2-Aa/H2-Ab1/H2-Eb1/Cyba/Tlr2/Stat1/H2-DMb2/Fcgr4/H2-DMb1/Ncf2/Ptpn6/Ifng
Cytosolic DNA-sensing pathway	9	0.001973	Cxcl10/Ccl5/Zbp1/Irf7/Mavs/Ddx58/Ifi202b/LOC100044068/Casp1

Supplemental Table 2. Enrichment analysis of genes altered in MRL/lpr+MSCT *vs.* MRL/lpr mice by GSEA with the KEGG (top) and Reactome (bottom) modules. A list of genes was generated with lower expression in MRL/lpr+MSCT than in MRL/lpr (≥ 1.5-fold change and *P* value < 0.05).

MRL/lpr + MSCT < MRL/lpr (KEGG)

Rnk	Pathways	P value	Altered genes
1	Antigen processing and presentationm	9.4E-18	H2-K1/H2-Ab1/H2-T23/H2-D1/B2m/H2-DMb2/Ciita/Tap1/H2-M2/H2-Aa/H2-Eb1/H2-DMb1/H2-T22/H2-T9/Cd74/H2-Q1/Cd8a/Tap2/ Ifi30/ Ifng/Tapbp/H2-Ob/Psme2/Cd4
2	Cell adhesion molecules (CAMs)	1.5E-17	H2-K1/H2-Ab1/H2-T23/Ptprc/Itgb7/Itgal/H2-D1/Sell/H2-DMb2/H2-M2/Cd2/Cd274/H2-Aa/Spn/H2-Eb1/H2-DMb1/H2-T22/H2-T9/H2-Q1/Pdcd1/Cd8a/Icam1/Ctla4/Siglec1/Cd6/Itgb2/Icos/Cd28/H2-Ob/Cd4/Cd22/Cd86
3	*Staphylococcus aureus* infection	4.7E-14	H2-Ab1/C1s1/Itgal/H2-DMb2/C1qc/H2-Aa/C1ra/C1qb/H2-Eb1/H2-DMb1/Ptafr/Cfh/Icam1/Itgb2/C3/H2-Ob/C4b/C1qa
4	Allograft rejection	9.1E-14	H2-K1/H2-Ab1/H2-T23/H2-D1/H2-DMb2/H2-M2/H2-Aa/H2-Eb1/H2-DMb1/H2-T22/H2-T9/H2-Q1/Ifng/Cd28/H2-Ob/Cd86/Gzmb
5	Graft-versus-host disease	9.1E-14	H2-K1/H2-Ab1/H2-T23/H2-D1/H2-DMb2/H2-M2/H2-Aa/H2-Eb1/H2-DMb1/H2-T22/H2-T9/H2-Q1/Ifng/Cd28/H2-Ob/Cd86/Gzmb
12	Phagosome	1.1E-10	H2-K1/H2-Ab1/H2-T23/H2-D1/H2-DMb2/Tap1/H2-M2/H2-Aa/Cybb/C1ra/Clec7a/H2-Eb1/H2-DMb1/H2-T22/H2-T9/H2-Q1/Mrc2/Tap2/Ncf4/Itgb2/C3/Tlr4/H2-Ob/Itga2/Cyba/Cd14
13	Cytokine-cytokine receptor interaction	2.2E-10	Ccl8/Cxcl10/Ltb/Cxcl13/Cxcl11/Ccr2/Il2ra/Ccl5/Il2rg/Il18r1/Il9r/Cxcr6/Tnfrsf25/Il2rb/Cxcr3/Cxcl16/Tnfrsf1b/Ccr9/Ifng/Cxcr4/Xcr1/Csf2rb/Csf1/Il7r/Ccr5/Cd27/Tnfrsf14/Ccr7/Ccl22/Osmr
18	Systemic lupus erythematosus	1.4E-07	H2-Ab1/C1s1/H2-DMb2/C1qc/H2-Aa/C1ra/C1qb/H2-Eb1/H2-DMb1/Ifng/Cd28/Hist1h4k/C3/H2-Ob/C4b/C1qa/Trim21/Cd86
19	Chemokine signaling pathway	2.2E-06	Ccl8/Cxcl10/Cxcl13/Cxcl11/Ccr2/Rac2/Ccl5/Cxcr6/Stat1/Cxcr3/Dock2/Itk/Cxcl16/Ccr9/Cxcr4/Xcr1/Ccr5/Ccr7/Ccl22/Hck/Adcy7
24	Jak-STAT signaling pathway	2.3E-04	Socs1/Il2ra/Irf9/Il2rg/Il9r/Stat1/Il2rb/Pim1/Ifng/Stat5a/Csf2rb/Il7r/Spry1/Osmr
27	Endocytosis	8.2E-04	H2-K1/Ldlr/H2-T23/Psd4/H2-D1/H2-M2/Il2ra/Il2rg/Il2rb/H2-T22/H2-T9/Pard6b/H2-Q1/Acap1/Cxcr4/Ccr5/Git2/Arf6

MRL/lpr + MSCT < MRL/lpr (Reactome)

Rnk	Description	P value	Altered genes
1	Adaptive Immune System	2.3E-21	H2-K1/H2-Ab1/Lcp2/H2-T23/Ifitm1/Psmb9/Itgb7/Psmb8/Cd3d/Cd3e /Itgal/H2-D1/H2-L/B2m/Sell/Cd19/H2-DMb2/Lck/Lat/Tap1/H2-M2/Cd274/H2-Aa/Cybb/Erap1/Ifitm3/Cd3g/Cd247/Cd79a/H2-Eb1/H2-DMb1/H2-T22/H2-T9/Cd74/H2-Q1/Pdcd1/Cd8a/Tap2/Itk/Syk/Ncf4/Icam1/Ctla4/Ifi30/Ripk2/Itgb2/Icos/Cd28/Grap2/Tapbp/C3/Tnfrsf14/H2-Ob/Psme2/Cyba/Cd4/Card11/Cd86
2	Interferon gamma signaling	2.9E-21	Gbp2/H2-K1/H2-Ab1/H2-T23/Irf1/Irf4/Gbp5/H2-D1/H2-L/B2m/Socs1/Ciita/H2-M2/Irf9/Trim30a/H2-Aa/Trim12c/Stat1/H2-Eb1/H2-T22/H2-T9/H2-Q1/Ptafr/Icam1/Ifi30/Ifng/Oas3/Irf8/Trim21/ Gbp2b
3	Interferon Signaling	9.7E-21	Gbp2/H2-K1/H2-Ab1/H2-T23/Irf1/Irf4/Gbp5/H2-D1/H2-L/Uba7/B2m/Socs1/Ciita/H2-M2/Irf9/Trim30a/H2-Aa/Trim12c/Stat1/H2-Eb1/H2-T22/H2-T9/H2-Q1/Ptafr/Icam1/Ifi30/Ifng/Ube2l6/Oas3/Irf8/Isg15/ Trim21/Gbp2b
13	Chemokine receptors bind chemokines	2.1E-09	Cxcl10/Cxcl13/Cxcl11/Ccr2/Ccl5/Cxcr6/Cxcr3/Cxcl16/Ccr9/Cxcr4/Xcr1/Ccr5/Ccr7/Ccl22

Supplemental Table 3. Oligonucleotide primers used for mRNA real-time PCR

Gene	Sense primer (5ˈ to 3ˈ)	Antisense primer (5ˈ to 3ˈ)
m*Ccl8*	GTAGACCCCACACAGAAGTGG	GGAGAACTTCCAGCTTTGGC
m*Cxcr3*	ATGGGGTCTCTGTCTGCTCT	TGAGGCGCTGATCGTAGTTG
m*Ccl3*	CAGCGAGTACCAGTCCCTTT	GCAGTGGTGGAGACCTTCAT
m*Ccl28*	AGTGGGTCAGGCGGGAATG	GAGGTTTGAAAAGCCACACACA
m*Cxcl9*	GGGACCACAGACTATTCCCC	GCCAATGCCTGGTGTGTAAC
m*Cxcl10*	TGAGAGACATCCCGAGCCAA	GAGGCAGAAAATGACGGCAG
m*Ccr1*	TTGTCCATGCTGTGTTTGCC	GGCAGGCATGGAAGCTAAGA
m*Ccr2*	GCCATCATAAAGGAGCCATACC	TGTGGTGAATCCAATGCCCT
m*Ccr8*	ATCCGACCTGCTCTTTGTCC	GGCCAGAGACCACCTTACAC
m*Ifng*	TGAATGTCCAACGCAAAGCA	CTGTTTTAGCTGCTGGCGAC
m*Ifitm3*	CCCCTTACCCCTTCATTCCTT	GCCGTAGGACATTGGGAGTA
m*Gapdh*	CCAGCTACTCGCGGCTTTA	GTTCACACCGACCTTCACCA
h*IFNG*	GAAAGTTGGGGGAGTGTGCT	GGGTCTCATCTAATGGGCCG
h*IFITM3*	TGCTGATCTTCCAGGCCTAT	AGCGTGTGAGGATAAAGGGC
h*CCL8*	TCCCAGGATCTGGTGCTTACT	AACCTCTCTGCTCCTCGGTG
h*GAPDH*	CCGCATCTTCTTTTGCGTCG	ATCCGTTGACTCCGACCTTC

391 **Supplemental Table 4. Key resources table**

Reagent or resource	Source	Identifier
**Antibodies**		
Anti-beta-actin (HRP-conjugate)	Abcam	Cat# ab20272
Anti-PSD-95	Abcam	Cat# ab2723
Anti-PSD-95	Proteintech	Cat# 20665-1-AP
Anti-Synaptophysin	Proteintech	Cat# 17785-1-AP
Anti-Synaptophysin	Synaptic Systems	Cat# 101 002
Anti-CD68	Bio-Rad	Cat# AB_322219
Anti-CD68	Proteintech	Cat# 66231-2-Ig
Anti-IBA-1	Wako	Cat# 019-19741
Anti-TMEM119	Abcam	Cat# ab209064
Anti-JAK1	Cell Signaling Technology	Cat# 3332
Anti-p-JAK1	Cell Signaling Technology	Cat# 3331
Anti-JAK2	Cell Signaling Technology	Cat# 3230
Anti-p-JAK2	Cell Signaling Technology	Cat# 3771
Anti-STAT1	Cell Signaling Technology	Cat# 9172
Anti-p-STAT1	Cell Signaling Technology	Cat# 9167
Horseradish peroxidase-conjugated goat anti-mouse IgG secondary antibody	Thermo Fisher Scientific	Cat# 31430
Horseradish peroxidase-conjugated goat rabbit IgG secondary antibody	Thermo Fisher Scientific	Cat# 31460
Alexa Fluor 555 goat anti-mouse IgG	Invitrogen	Cat# A 21422
Alexa Fluor 555 goat anti-rabbit IgG	Invitrogen	Cat# A21429
Alexa Fluor 555 donkey anti-goat IgG	Invitrogen	Cat# A21432
Alexa Fluor 488 goat anti-rabbit IgG	Invitrogen	Cat# A11008
Alexa Fluor 488 donkey anti-mouse IgG	Invitrogen	Cat# A21202
APC anti-mouse CD3e	BD Biosciences	Cat# 561826
FITC anti-mouse CD4	BD Biosciences	Cat# 561828
BV510 anti-mouse CD8α	BD Biosciences	Cat# 563068
BV786 anti-mouse IFN-γ	BD Biosciences	Cat# 563773
BV605 anti-human CD3	BD Biosciences	Cat# 564712
FITC anti-human CD4	BD Biosciences	Cat# 550628
PerCP-Cy5.5 anti-human CD8	Biolegend	Cat# 344710
ACP anti-human IFN-γ	BD Biosciences	Cat# 562017
**Chemicals, peptides, and recombinant proteins**		
ProLong Gold Antifade Mountant	Invitrogen	Cat# P36930
PLX5622	Med Chem Express	Cat# HY-114153
Pristane	Sigma-Aldrich	Cat# P2870
Mineral oil	Sigma-Aldrich	Cat# M5904
Human T-Activator CD3/CD28	eBioscience	Cat# 11161D

Ruxolitinib	Selleck	Cat# S1378
Fludarabine	Selleck	Cat# S1491
Recombinant mouse CCL8 protein	BioLegend	Cat# 581704
Mouse IFN-γ protein	R&D Systems	Cat# 485-MI
IFN-γ blocking antibody	R&D Systems	Cat# MAB10262
Rat IgG2b isotype control	R&D Systems	Cat# 141945
DNase I	Sigma-Aldrich	Cat# D5025-15KU
Collagenase type IV	Sigma-Aldrich	Cat# 11088858001
**Experimental models:**		
Mouse: MRL/MpJ-*Fas*^{lpr}	Shanghai Lingchang Biotechnology Corporation	The Jackson Laboratory: stock #006825
Mouse: MRL/MpJ	Shanghai Lingchang Biotechnology Corporation	The Jackson Laboratory: stock # 000486
Mouse: *Syn1*^{Cre}	Slac Laboratory Animal Center	The Jackson Laboratory: stock # 003966
Mouse: *Ccl8*^{floxed}	Cyagen Biosciences	stock # CKOCMP-01361-Ccl8
Mouse: C57BL/6J	Model Animal Research Center of Nanjing University	N/A
**Software and algorithms**		
Prism 6 Software	GraphPad	https://www.graphpad.com/scientific-software/prism/
ImageJ Software	NIH	https://imagej.nih.gov/ij/
Imaris 8.3.1	Bitplane	http://www.bitplane.com/imaris
TopScan Software	CleverSys	http://cleversysinc.com/products/software/topscan/
FlowJo software V10	Tree Star	
**Other**		
FD Rapid GolgiStain Kit	FD Neurotechnologies	Cat# PK401
HiScript III RT SuperMix for qPCR Kit	Vazyme	Cat# R323-01
ChamQ SYBR qPCR Master Mix	Vazyme	Cat# Q341-02
CD11b (Microglia) MicroBeads	Miltenyibiotec	Cat# 130-093-636
Bradford Protein Detection Kit	Keygen	Cat# KGA801-804
Percoll	GE Healthcare	Cat# 17-0891-09
EasySep™ Human CD4^{+} T-Cell Isolation Kit	STEMCELL	Cat# 17952
EasySep™ Human Naive CD4^{+} T-Cell Isolation Kit	STEMCELL	Cat# 19555
EasySep™ Mouse CD4^{+} T-Cell Isolation Kit	STEMCELL	Cat# 19852
Mouse anti-dsDNA ELISA Kit	FUJIFILM	Cat# 631-02699
Mouse anti-CCL8 ELISA Kit	Abcam	Cat# ab203366
Human anti-IFN-γ ELISA Kit	FCMRCS	Cat# FMS-ELH035
Human anti-CCL8 ELISA Kit	Abcam	Cat# ab223856
RNA FISH staining Kit	GenePharma	Cat# F22201

392

Supplemental Table 5. Patient data for all samples used in this study

Serum and PBMC samples

Internal Reference	Age (year)	Sex	SLEDAI	Clinical manifestations
Control Group				
n = 11	36.8 (Average)	M (3), F (8)	-	-
SLE Group				
SLE 1	40	F	13	C
SLE 2	47	M	22	C、V
SLE 3	43	M	8	C
SLE 4	30	M	20	C、V
SLE 5	28	F	24	LN、C
SLE 6	32	F	20	LN、C
SLE 7	28	F	20	LN、C
SLE 8	28	F	18	LN、C
SLE 9	43	F	15	A、C
SLE 10	52	F	8	C、LN
SLE 11	30	F	26	LN、C、A
SLE 12	58	F	16	LN
SLE 13	34	F	11	C
SLE 14	33	F	10	C
SLE 15	23	F	20	C

A, Arthritis; C, Cytopenia; F, Febrile; LN, Lupus nephritis; NP, Neuropsychiatric; V, Vasculitis.

CSF samples

Internal Reference	Age (year)	Sex	SLEDAI	Clinical manifestations
Control Group				
n = 6	30.7 (Average)	M (2), F (4)	-	-
Non-NPSLE Group				
non-NPSLE 1	18	F	2	F
non-NPSLE 2	42	M	5	LN
non-NPSLE 3	15	F	13	F、C
non-NPSLE 4	48	F	17	LN
non-NPSLE 5	26	F	8	A
non-NPSLE 6	63	F	10	A、C、LN
non-NPSLE 7	53	F	7	LN
non-NPSLE 8	13	M	2	F
non-NPSLE 9	32	F	8	F、C
NPSLE Group				
NPSLE 1	30	F	24	F、C、NP
NPSLE2	42	F	23	A、NP
NPSLE 3	40	F	16	A、C、V、NP
NPSLE 4	43	F	12	A、C、NP
NPSLE 5	22	F	27	F、C、LN、NP
NPSLE 6	14	F	8	F、NP
NPSLE 7	57	M	12	C、LN、NP
NPSLE 8	48	F	20	A、C、LN、NP
NPSLE 9	54	F	26	A、C、NP
NPSLE 10	27	F	18	A、C、NP
NPSLE 11	31	M	12	F、C、NP
NPSLE 12	34	F	16	NP
NPSLE 13	16	F	14	F、NP
NPSLE 14	56	F	4	F、NP

A, Arthritis; C, Cytopenia; F, Febrile; LN, Lupus nephritis; NP, Neuropsychiatric; V, Vasculitis.

398 **Supplemental Table 6. O-link data**

Panel	Assay	UniProt ID	OlinkID	Missing Data freq.
Olink INFLAMMATION	IL8	P10145	OID00471	0%
	VEGFA	P15692	OID00472	0%
	CD8A	P01732	OID05124	0%
	CDCP1	Q9H5V8	OID00476	0%
	IL7	P13232	OID00478	0%
	OPG	O00300	OID00479	0%
	LAP TGF-beta-1	P01137	OID00480	0%
	uPA	P00749	OID00481	0%
	IL6	P05231	OID00482	0%
	MCP-1	P13500	OID00484	0%
	CXCL11	O14625	OID00486	0%
	TRAIL	P50591	OID00488	0%
	CXCL9	Q07325	OID00490	0%
	CST5	P28325	OID00491	0%
	OSM	P13725	OID00494	0%
	CXCL1	P09341	OID00496	0%
	CCL4	P13236	OID00498	0%
	CD6	P30203	OID00499	0%
	SCF	P21583	OID00500	0%
	IL18	Q14116	OID00501	0%
	TGF-alpha	P01135	OID00503	0%
	MCP-4	Q99616	OID00504	0%
	CCL11	P51671	OID00505	0%
	TNFSF14	O43557	OID00506	0%
	FGF-5	P12034	OID00509	0%
	MMP-1	P03956	OID00510	0%
	LIF-R	P42702	OID00511	0%
	CCL19	Q99731	OID00513	0%
	IL-10RB	Q08334	OID00515	0%
	IL-18R1	Q13478	OID00517	0%
	PD-L1	Q9NZQ7	OID00518	0%
	CXCL5	P42830	OID00520	0%
	HGF	P14210	OID00522	0%
	IL-12B	P29460	OID00523	0%
	MMP-10	P09238	OID00527	0%
	CCL23	P55773	OID00530	0%
	CD5	P06127	OID00531	0%
	CCL3	P10147	OID00532	0%
	Flt3L	P49771	OID00533	0%
	CXCL6	P80162	OID00534	3%
	CXCL10	P02778	OID00535	6%
	4E-BP1	Q13541	OID00536	6%
	SIRT2	Q8IXJ6	OID00538	10%
	CCL28	Q9NRJ3	OID00539	10%
	IFN-gamma	P01579	OID05547	13%

	DNER	Q8NFT8	OID01213	13%
	CD40	P25942	OID00542	13%
	FGF-19	O95750	OID00545	13%
	MCP-2	P80075	OID00549	16%
	CCL25	O15444	OID00551	16%
	CX3CL1	P78423	OID00552	19%
	TNFRSF9	Q07011	OID00553	23%
	TWEAK	O43508	OID00555	23%
	ADA	P00813	OID00560	26%
	TNFB	P01374	OID00561	32%
	CSF-1	P09603	OID00562	35%
	TNF	P01375	OID05548	39%
	IL10	P22301	OID00528	39%
	IL-24	Q13007	OID00524	42%
	IL13	P35225	OID00525	45%
	ARTN	Q5T4W7	OID00526	45%
	IL-15RA	Q13261	OID00514	55%
	TRANCE	O14788	OID00521	55%
	Beta-NGF	P01138	OID00519	58%
	IL-22 RA1	Q8N6P7	OID00516	65%
	AXIN1	O15169	OID00487	71%
	SLAMF1	Q13291	OID00502	77%
	FGF-21	Q9NSA1	OID00512	81%
	FGF-23	Q9GZV9	OID00507	81%
	IL-10RA	Q13651	OID00508	84%
	TSLP	Q969D9	OID00497	84%
	IL-20RA	Q9UHF4	OID00489	84%
	IL-2RB	P14784	OID00492	90%
	IL-1 alpha	P01583	OID00493	90%
	IL2	P60568	OID00495	90%
	MCP-3	P80098	OID00474	90%
	GDNF	P39905	OID00475	94%
	CD244	Q9BZW8	OID00477	94%
	IL-17C	Q9P0M4	OID00483	94%
	IL-17A	Q16552	OID00485	94%
	IL-20	Q9NYY1	OID00537	94%
	EN-RAGE	P80511	OID00541	94%
	IL33	O95760	OID00543	100%
	LIF	P15018	OID00547	100%
	IL4	P05112	OID00546	100%
	NT-3	P20783	OID00554	100%
	CCL20	P78556	OID00556	100%
	ST1A1	P50225	OID00557	100%
	STAMBP	O95630	OID00558	100%
	IL5	P05113	OID00559	100%
	CASP-8	Q14790	OID00550	100%
	NRTN	Q99748	OID00548	100%
