# Supplementary figures and images for: MSC transplantation ameliorates depression in lupus by suppressing Th1 cell–shaped synaptic stripping
Source: JCI Insight. 2025 Mar 6;10(8):e181885. doi: 10.1172/jci.insight.181885 (PMC12016924; doi:10.1172/jci.insight.181885)

Full unedited gel for

Fig. 4I

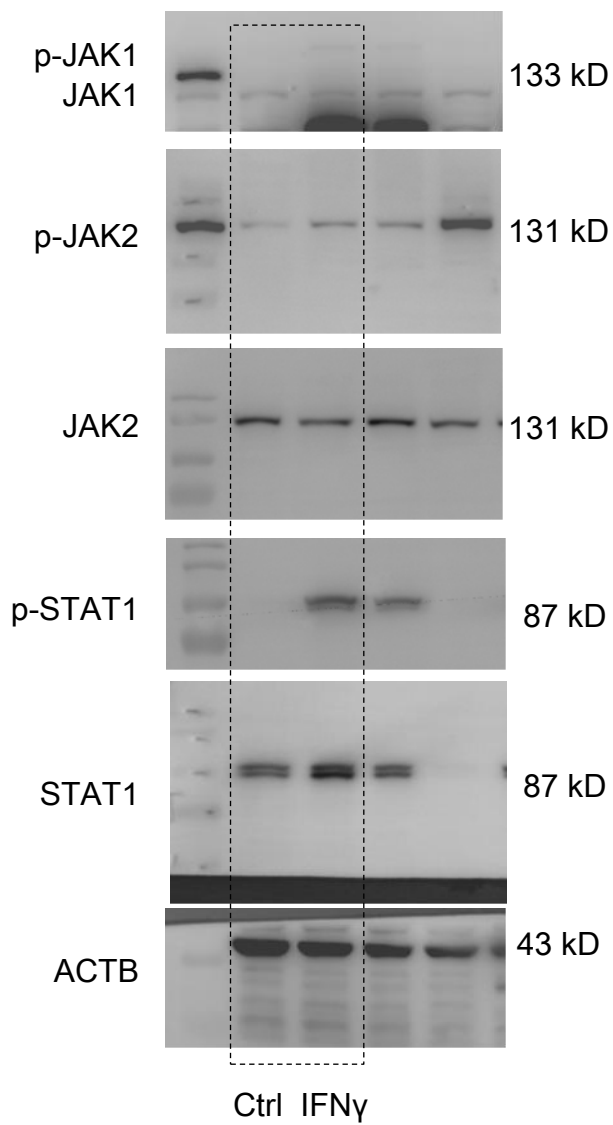

Fig. 6E

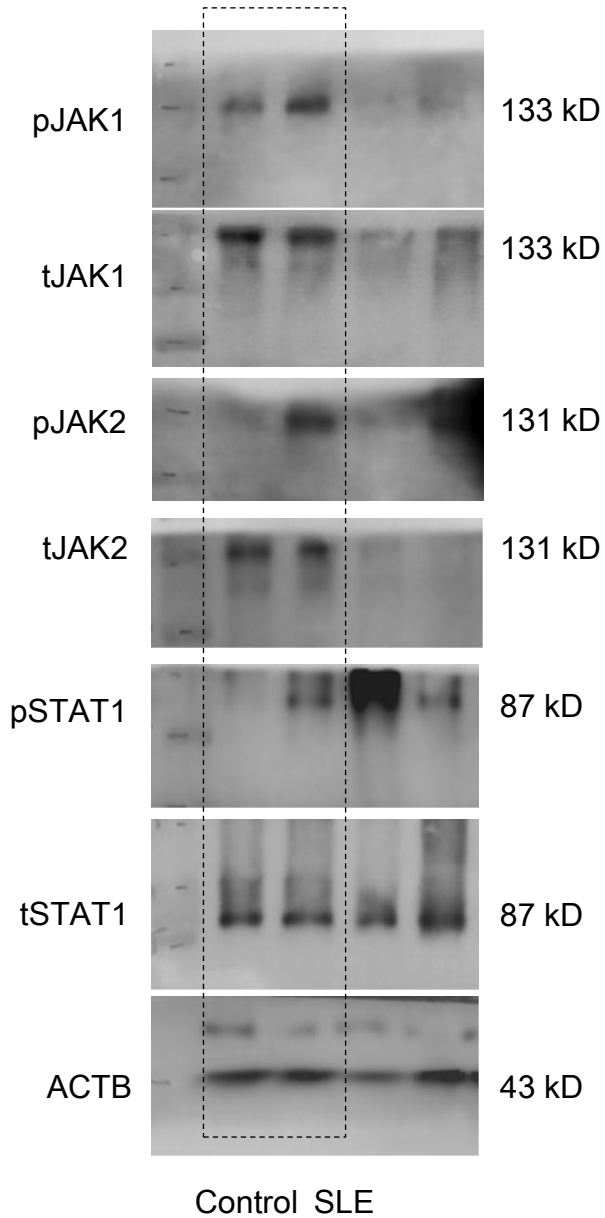

Supplement: Unedited blot and gel images [file jciinsight-10-181885-s145.pdf]
